# DNA-Lipid Nanodiscs with a Polyethylene Glycol Interface

**DOI:** 10.1021/jacs.6c03471

**Published:** 2026-05-04

**Authors:** Soumya Chandrasekhar, Christopher Maffeo, Sanjai Karanth, Rachel Bricker, Joy Kabuga, Diana Patricia Nunes Gonçalves, Aleksei Aksimentiev, Thorsten L. Schmidt

**Affiliations:** † Department of Physics, 4229 Kent State University, Kent, Ohio 44242, United States; ‡ Advanced Materials and Liquid Crystal Institute, Kent State University, Kent, Ohio 44242, United States; § Department of Physics, 14589 University of Illinois at Urbana–Champaign, Urbana, Illinois 61801, United States; ∥ Beckman Institute for Advanced Science and Technology, University of Illinois at Urbana–Champaign, Urbana, Illinois 61801, United States; ⊥ Department of Bioengineering, University of Illinois at Urbana–Champaign, Urbana, Illinois 61801, United States; # Department of Chemistry, Kent State University, Kent, Ohio 44242, United States

## Abstract

Nanoscale bilayer mimetics such as protein or polymer-based nanodiscs are versatile tools to study the physical chemistry of lipid bilayers or the structures and functions of membrane proteins. Here, we introduce DNA-lipid nanodiscs (DLNs) in which the interface between hydrophobic lipids and the charged DNA is mediated through amphiphilic poly­(ethylene)­glycol (PEG). For this, we modified oligonucleotides with PEG and hybridized them to a single-stranded ring to form functionalized minicircles with a well-defined diameter. The center of these minicircles can be filled with a lipid bilayer through addition of detergent-solubilized lipids followed by detergent removal. Simulations reveal that the methylene groups in PEG form dynamic interactions with the acyl chains of lipids, effectively shielding the hydrophobic mismatch. As proof of concept toward incorporation of complex membrane proteins, we inserted the biotinylated transmembrane domain of synaptobrevin into these nanodiscs and bound them to streptavidin-modified quantum dots as a marker for successful incorporation. We envision these atomically precise, modular DNA scaffolds to be widely applicable in future studies of membrane proteins and nanoscale lipid membranes.

## Graphical abstract

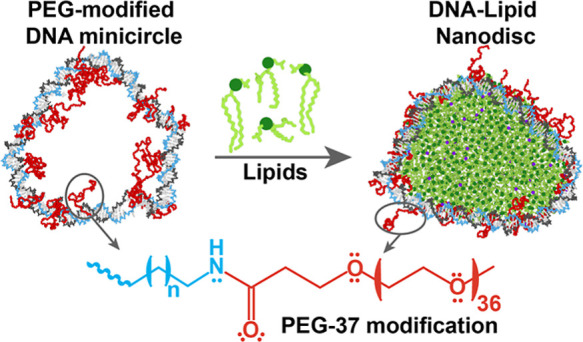

## Introduction

Lipid membranes and membrane proteins are critical components of all cells, playing vital roles in cellular structure, function and communication.^1^ Several nanoscale bilayer mimetics including liposomes,^2,3^ bicelles^4^ and nanodiscs^5–7^ have been developed to study the structure and function of membrane proteins in their native lipid environment. Nanodiscs^8^ are nanoscale, discoidal particles where the rim of the lipid bilayer is limited and stabilized by amphipathic proteins (membrane scaffolding proteins—MSPs),^9^ peptides (peptidiscs),^10–12^ polymers,^13^ or *S*-alkyl-modified DNA.^14^ MSP nanodiscs consist of an apolipoprotein (apo) A-I belt that wraps around the lipid bilayer and typically ranges from 7 to 17 nm in diameter.^15^ Styrene-maleic acid (SMALPs)^13^ and various other amphiphilic copolymers such as Ultrasolute Amphipols,^16^ poly­(acrylic acid-*co*-styrene) (AASTY),^17^ diisobutylene-*alt*-maleic acid (DIBMA)^18^ can solubilize membranes.

In established bilayer mimetic systems, it is difficult to precisely predetermine the nanodisc size, to add additional functional groups or to control the position of such functionalizations on the belt. Structural DNA nanotechnology on the other hand provides an approach for the modular design and customization of nanostructures with precise control over size and the positions where functionalizations are introduced.^19^ Being a polyanion, DNA can only form interfaces with membranes by adding hydrophobic modifications to its backbone or nucleobases.^20^ Hydrophobic DNA modifications include cholesterol,^21,22^ tocopherol,^23^ lipids,^24,25^ porphyrins,^26–28^ pyrene,^29^ and *S*-alkylated phosphorothioates.^14,30^ Such modified DNA oligonucleotides were used to create DNA nanopores^30,31^ DNA-lipid nanodiscs,^14,32^ to mimic enveloped virus particles,^24^ or to deform,^33–35^ pattern,^36,37^ fuse^38,39^ and control budding^40^ of membranes. Previously, we synthesized a DNA-lipid nanodisc (DLN) from a double stranded (ds) DNA minicircle formed from a covalently closed single-stranded (ss) circle and seven identical, complementary, modified oligonucleotides, in which *S*-alkyl phosphorothioates enable interactions with lipid membranes.^14^ This system generated nanodiscs of uniform size which was dictated by the length of the ss circle. Subsequent coarse-grained molecular dynamics simulations suggested that greater charge neutralization could produce stronger interactions with lipid membranes.^41^ However, we recently found that extensively *S*-alkylated ds DNA is structurally and thermally destabilized^42^ and that this is not a viable route to further increasing DLN stability.

Thus, we seek to explore alternative chemical modifications of DNA to interface with lipid bilayers that do not involve extensive charge neutralization or lead to aggregation. Previously, we found that DNA origamis that are coated with poly­(ethylene) glycol (PEG) block copolymers are protected from enzymatic degradation.^43,44^ Moreover, PEG-modified origamis can even be quantitatively transferred across the phase boundary of a two-phase solution into organic solvents including chloroform, in which DNA is usually not soluble.^45^ The phase transfer takes place because the surface of the origamis is covered with PEG, which is an amphiphilic molecule that is even better soluble in chloroform than in water. As lipids are also commonly solubilized in chloroform, we hypothesized that the ethylene groups of the amphiphilic PEG molecules can provide an interface with the hydrophobic acyl chains of lipid membranes of a DLN. In this work, we covalently modified DNA circles with PEG, optimized the length and number of PEG modifications, incorporated a transmembrane peptide and analyzed the resulting complexes and interfaces with atomistic molecular dynamic simulations.

## Results and Discussion

### Oligonucleotide PEGylation

For this, we designed a 21-base oligonucleotide with up to three reactive amine modifications; one each at the 5′ and 3′ ends, and one at a modified thymidine (Figure 1A). The amino groups were reacted with PEG-37 NHS esters (molecular weight ∼2 kDa) through a nucleophilic substitution resulting in a stable amide bond. In separate experiments, we also conjugated shorter PEG-3- and PEG-13-NHS esters or an oligonucleotide with only one amine modification to investigate the respective effects of length and number of PEG chains in stable nanodisc formation (Figure S1). The PEG-oligonucleotide was purified by reverse-phase high performance liquid chromatography (RP-HPLC) (Figure S2) and characterized by high resolution liquid chromatography–mass spectrometry (LC–MS) (Figure S3). In denaturing polyacrylamide gel electrophoresis of the oligonucleotides (PAGE, Figure 1B), the addition of PEG reduces their electrophoretic mobility.

**Figure 1:**
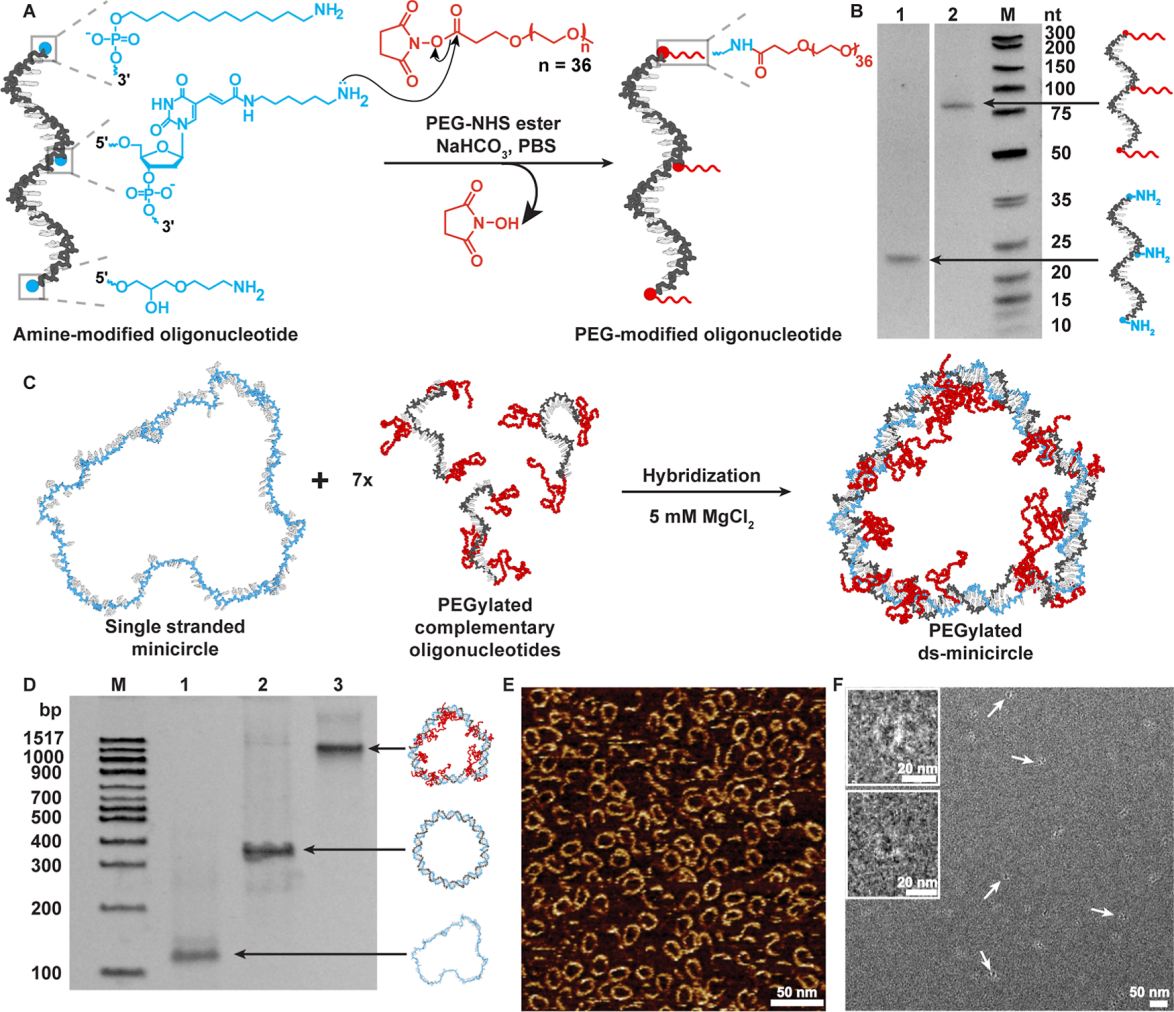
Synthesis and characterization of PEG-modified DNA minicircles. (A) A 21-mer oligonucleotide with three amine modifications was modified with PEG-NHS esters. (B) Denaturing PAGE of amine oligonucleotide, before and after reaction with PEG-37-NHS ester. (C) Hybridization of the circular single-stranded scaffold with seven identical complementary PEG-modified strands form a double-stranded (ds) circle. Shown are snapshots from MD simulations that illustrate the different flexibility of the polymers and ds DNA. (D) Native PAGE of the different circles. (M) Marker, (1) ss DNA circle/scaffold, (2) ds circle formed with seven complementary unmodified oligonucleotides, (3) ds circles with seven complementary oligonucleotides, each modified with three PEG-37. AFM (E) and TEM (F) images of PEG-modified ds circles. Scale bar: 50 nm. Inset: zoomed-in image of ds minicircles.

### Formation of PEGylated Minicircle

We then hybridized the PEGylated oligonucleotides with a 147 nt (nucleotide) ss-minicircle scaffold that we prepared by splint ligation of a long strand (Figure 1C).^14^ The scaffold sequence contains seven repeats of an intrinsically bent A-tract motif^64^ that is complementary to the PEG-modified oligonucleotides. The ds-minicircles thus formed have an outer diameter of about 18 nm and an inner diameter of 14 nm (calculated using 0.34 nm/bp step) and are 2 nm thick. In a native polyacrylamide gel (PAGE), the modified ds circles form a sharp band with a lower electrophoretic mobility than ds minicircles without PEG modifications (Figure 1D). In atomic force microscopy (AFM) images (Figure 1E), and transmission electron microscopy (TEM) images (Figure 1F), the ds-PEG-minicircles appear intact and circular. The contrast of rings is higher in AFM than in TEM as DNA is not stained well in TEM. Moreover, the sample density is higher in AFM as rings adhere better to the positivized mica substrate than to TEM grids.

Note that this chemical coupling reaction does not neutralize charges in the DNA backbone as in *S*-alkylation of phosphorothioates^14,31^ that reduces their melting temperature (*T*_m_) and can destabilize hybridization.^42^ Despite the large added molecular mass, PEG-modification only lowered the melting temperature *T*_m_ with the ss minicircle by 4–5 °C (Figure S4).

### Synthesis of PEGylated DNA-Lipid Nanodiscs

To reconstitute a lipid bilayer within the PEGylated minicircle (Figure 2A), we mixed them with detergent-solubilized lipids and removed the detergents through a spin column. While attempts with zwitterionic detergents such as CHAPS or nonionic detergents like Tween-20 did not result in DLNs, DLNs readily formed with positively charged detergents such as dodecyl trimethylammonium bromide (DTAB). This suggests that electrostatic interactions between mixed micelles and the negatively charged phosphate backbone of DNA are crucial for the formation of DLNs. The optimized lipid formulation contained 89% DMPC (1,2-dimyristoyl-*sn*-glycero-3-phosphocholine), which has a zwitterionic headgroup, 10% DMTAP (1,2-dimyristoyl-3-trimethylammonium-propane) with a cationic headgroup and 1% of the fluorescent lipid Topfluor-PC (TfPC). 10% cationic lipid was found to be necessary for DLN formation (Figure S5) due to favorable electrostatic interactions with the phosphate backbone (Figure 3E–G). The detergent-solubilized lipids were mixed with dsDNA at a ratio of dsDNA/lipid = 1:450, which was calculated to fill the area inside the DNA minicircles. A TEM image of unpurified DLNs after the detergent removal step (Figure 2B) reveals the successful synthesis of nanodiscs with a better imaging contrast than the empty ds-PEG-rings (Figure 1F). For an image of an area also showing a liposome and for further details on imaging, see Figure S6. A control detergent removal experiment without PEGylated ds-minicircles was conducted, in which only liposomes formed as expected (Figure 2C).

**Figure 2:**
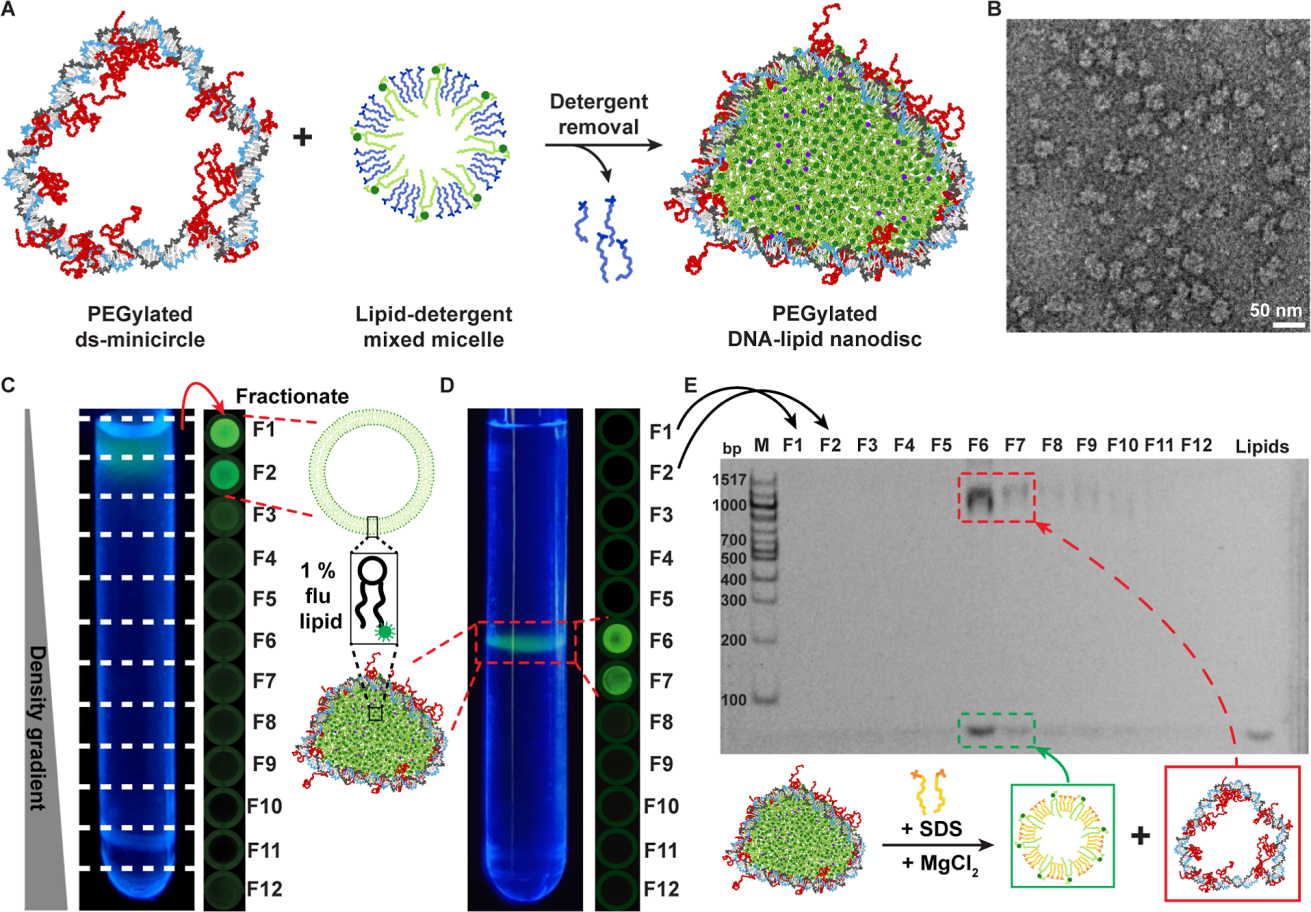
Synthesis and characterization of PEGylated DNA-lipid nanodiscs. (A) Lipid reconstitution in ds minicircles is achieved by adding lipid-detergent mixed micelles followed by detergent removal. (B) TEM image of ds minicircles with a lipid-filled interior. (C) Isopycnic ultracentrifugation of liposomes containing 1% fluorescent TfPC that were prepared by detergent removal without PEGylated DNA minicircles. The content of the ultracentrifugation tubes (left) was fractionated into the wells of a 96-well plate and imaged in a fluorescence scanner (right), indicating liposomes in the upper fractions (F1–F2). (D) Isopycnic centrifugation of DLNs showing lipids in F6–F7. (E) SDS PAGE analysis of the fractions taken from (D) confirms colocalization of PEGylated ds minicircle (red) and lipids (green) in fractions 6 and 7.

### Characterization of DLNs

Liposomes and DLNs were analyzed and purified through isopycnic ultracentrifugation in a density gradient made with iodixanol and buffer. Liposomes have a low density due to their large buffer-filled interior and float up to the top of the gradient (Figure 2C) whereas empty ds-PEG-rings stay at the bottom of the gradient (Figure S7) due to their high density. DLNs float up toward the middle, until their density matches that of the surrounding gradient. Under UV or blue light illumination, the Topfluor-PC in the lipid formulation fluoresces green. Additionally, the content of the ultracentrifugation tubes was fractionated into 96-well plates and imaged in a fluorescence scanner. While liposomes appeared in fractions 1 and 2, the lipids of DLNs were majorly found in fractions 6 and 7 (Figure 2C,D).

To prove the colocalization of DNA with the lipids in DLNs, we loaded aliquots of all fractions into a polyacrylamide gel that contained MgCl_2_ to stabilize dsDNA and the anionic detergent SDS that solubilizes the lipids into mixed micelles. DNA and lipids are therefore separated in the gel, but ds-rings remain intact. After gel electrophoresis, the gel was stained with the DNA stain SYBR gold.^65^ In this SDS gel, both ds-rings and lipids appeared in fractions 6 and 7 indicating stable complexes between the molecules (Figure 2E). A control lipid reconstitution experiment with ds-rings that were not PEG modified did not form DLNs (Figure S8), indicating that the PEG-37 modifications are necessary to hold a lipid bilayer. Staining the lipids with Nile red as an alternative to adding fluorescent lipids to the formulation failed due to strong nonspecific interactions of the dye with iodixanol in the density gradient (Figure S9).

### Optimizing PEG Content

Next, we reduced the number of PEG-37 modifications from three to one per oligonucleotide or seven PEG-37 per ds-ring to test how many PEG residues are necessary to hold the lipids in the ring, and no stable nanodiscs were observed for seven PEG modifications (Figure S10). Likewise, DLNs did not form with ds-circles containing shorter PEG-3 modifications and only inefficiently with PEG-13 chains (Figure S11), underlining the importance of longer PEG chains to stabilize the rim of the lipid bilayer.

Inspired by this result, we conjugated DNA to even longer PEG-5000 with an average molecular weight of 5 kDa. Nanodiscs only formed when the number of modifications per ring was reduced from 21 to 7 (Figure S12). We hypothesize that the much longer and bulkier PEG-5000 chains occupy more of the center of the ring and act like an entropic brush that prevents the incorporation of lipids when DNA is extensively modified. These results indicate that the optimal functionalization is 21 PEG-37 chains per ds-minicircle or 1.5 PEG-37 modifications per helical turn.

### Further Optimizations

Double-stranded DNA is much stiffer than single-stranded DNA, MSP or SMA scaffolds. We therefore incorporated seven 2 nt ss-gaps to create flexible joints to our minicircles. We hypothesized that these ss-gaps will cause the ring to be more compacted^66^ when empty, potentially facilitating nucleation of lipid mixed micelles during the lipid reconstitution. However, results were indistinguishable from experiments without gaps (Figure S13). We also identified that buffers used for the synthesis of other membrane mimetics (MSP, SMALP) are also suitable for the formation of the PEG-DLNs (Figure S14). However, high-salt buffers are detrimental to nanodisc formation (Figure S14) and thus we carried out DLN synthesis in buffers containing only 3 mM Mg^2+^, which is sufficient to stabilize the dsDNA without affecting the synthesis of the DLNs.

### MD Simulations of DLNs

Complementing the experimental characterization of DLNs, we constructed an all-atom system consisting of a patch of lipid bilayer surrounded by an idealized DNA ring functionalized with 1.5 PEG molecules per DNA turn to understand what molecular interactions take place at the interface between the lipid bilayer and the DNA. In addition, we partially neutralized the DNA-lipid interface with some cationic DTAB detergents to test if DTAB might remain bound to the negatively charged phosphates at the DNA-lipid interface after the detergent removal step in experiments, potentially stabilizing the DNA-lipid interface. The experimental synthesis involving detergent removal takes seconds to minutes and is therefore too slow to be simulated atomistically. Our simulations therefore concentrate on the final state of the nanodiscs and molecular interactions therein.

The system was submerged in ∼100 mM Na_2_SO_4_ and ∼6 mM MgCl_2_ electrolyte. During the first ∼120 ns of simulation, the non-hydrogen lipid atoms were harmonically restrained about their initial coordinates, and the DNA configuration was restrained via a stiff elastic network while the ions, DTAB, and PEG molecules were free to equilibrate. As restraints were gradually released, the entire DNA ring translated toward the upper leaflet of the bilayer (Figure 3A). After the restraints were released, the simulation was extended to 1 μs. We note that this time scale approximately matches the time required for each lipid type to diffuse a distance matching the interlipid spacing as determined by direct observation of the mean square displacement of lipids of each species, see Experimental Methods for details. The bilayer remained roughly circular whereas the DNA and PEG fluctuated extensively (Figure S15A; Movie 1). For comparison, an idealized bilayer patch was simulated without the DNA scaffold and also remained stable during the 1000 ns simulation (Movie 2). Note that in experiments such patches would fuse into lipid vesicles without additional stabilization of the edges.

Compared to the bare bilayer, the DNA-supported bilayer was marginally thinner and denser on average over the final 750 ns of simulation (Figure S15A,B). Moreover, the DNA, positioned on the upper leaflet, evidently induces a slight curvature (>100 nm radius) away from the DNA (Figure S15C), though the differences in the average structure of the core (4 nm cylindrical region) of the nanodisc-supported bilayer and bare bilayer are negligible. However, the edges of the bilayers are distinct: the supported bilayer exhibited ∼30% fewer lipid headgroups along the edge than the bare bilayer (Figure S15D). The reduction of lipids along the edges indicates that the PEG polymers function as designed, and 45% of the surface area of the lipid tails is occluded by PEG (Figure 3B), see Experimental Methods for details. Moreover, the total lipid tail surface area in the DLN is reduced to ∼75% of that exposed in the bare bilayer (16,400 vs 21,500 Å^2^), despite their identical lipid compositions, indicating that the DNA scaffold reduces solvent-accessible crypts between headgroups. Most molecular interactions occur between lipid tails and the methylene in PEG (Figure 3C), which are abundant in both molecules and are the main factor stabilizing the DNA-PEG nanodiscs. Normalizing by the total number of atom pairs for each type of contact (atom pairs within 4 Å), the contacts between PEG oxygen and lipid choline are the most probable, followed by glycerol–glycerol interactions involving the linker regions (Figure 3D). Strikingly, the interactions with lipid phosphates are quite rare in both absolute and relative senses.

DLN synthesis was only successful using 10% of cationic lipids (DMTAP) suggesting an electrostatic interaction between DNA scaffold and the charged lipids in the bilayer. In simulations, we indeed observed a ∼50% enrichment of the lipid fraction of charged DMTAP near the DNA on the upper leaflet, and an even greater depletion of DMTAP in the same area on the opposing leaflet (Figure 3E), indicating migration of DMTAP toward the DNA. Toward the end of the simulation, DMTAP was enriched in the central region of the bilayer of the lower leaflet, which might be attributed to electrostatic interactions across the periodic boundary of the system. DMTAP lipids flipped almost exclusively from lower to upper leaflet (Figure 3F,G), with approximately five migrating during the trajectory against a background of ∼45 DMPC lipids flipping in each direction.

**Figure 3:**
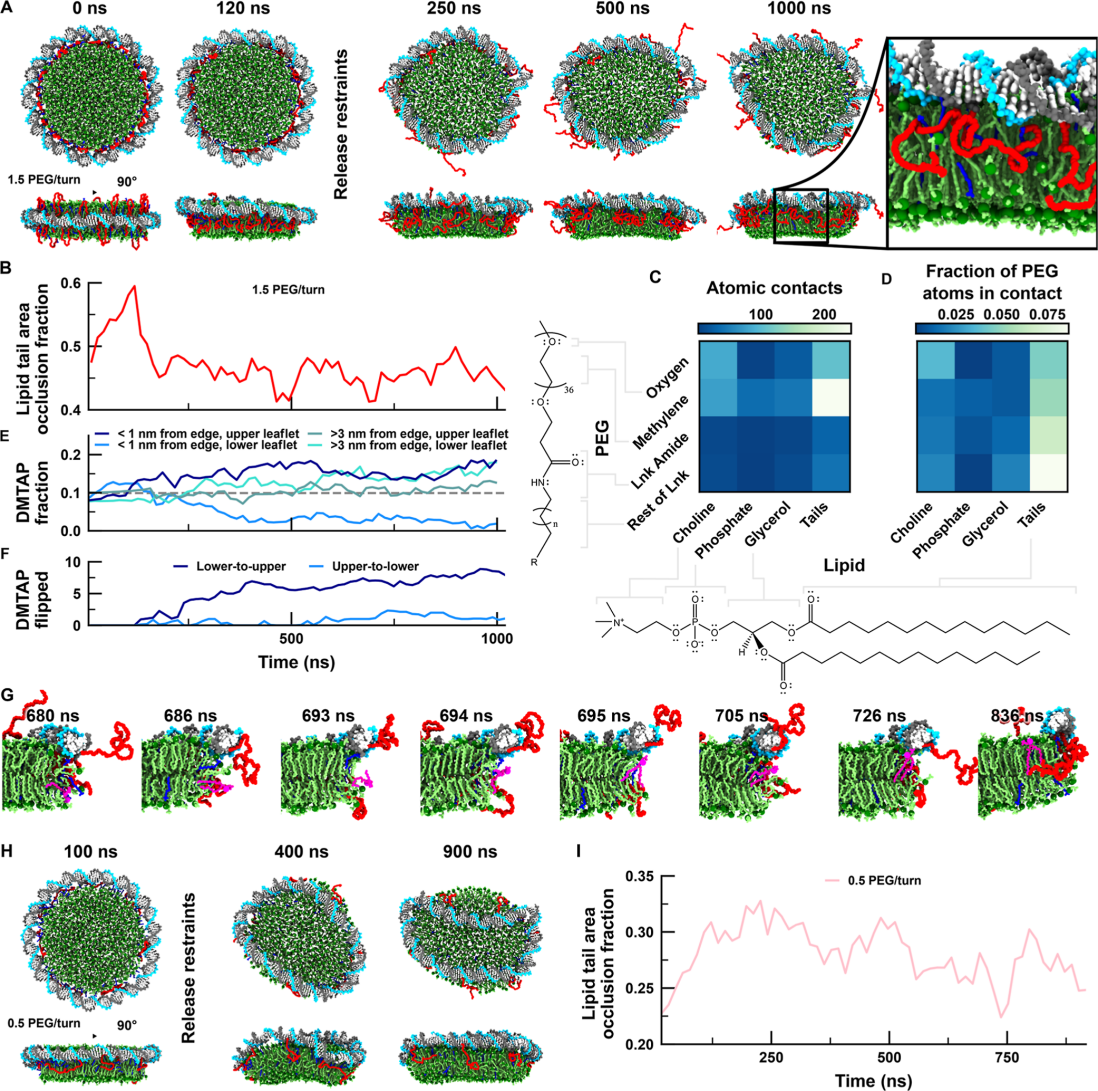
All-atom molecular dynamics simulations of PEGylated DLNs. (A) Snapshots of the restrained equilibration and unrestrained all-atom simulation trajectory of a DLN with 1.5 PEG-37 per DNA turn (21 total). (B) Fraction of lipid tail surface area occluded by PEG and DTAB in the DLN. The lipid tail area is calculated using a 2 Å probe radius; see Experimental Methods for details. (C,D) Contacts between lipid and PEG motifs, including linkers that tether PEG to the DNA. Both the number of contacts averaged over 250–1000 ns (C) and fraction of possible pairs forming a contact during the same interval (D) are depicted. (E) Ratio of DMTAP to all lipids, excluding DTAB, in four different regions of a DLN. The distance from the bilayer edge was computed as the lipid C2 atom distance from a 1 nm thick slice of the molecular surface representing the bilayer after projection into the plane of the bilayer, see Experimental Methods for details. The dashed gray line indicates the average DMTAP fraction in the system. A lipid was classified as being in the upper (lower) leaflet if its C2 atom was located above (below) the plane of the bilayer bisecting its center of mass. (F) Number of DMTAP lipids initially observed in the lower (upper) leaflet later observed in the upper (lower) leaflet. (G) Snapshots illustrating the flipping of a DMTAP lipid (pink). (H) Snapshots of an instable DLN with only seven instead of 21 PEG modifications (=0.5 PEG per helical turn). (I) Lipid tail area occlusion fraction as defined in panel B for the 0.5 PEG/turn system. All plotted time series were filtered with a 15 ns block average.

In contrast, the smaller cationic DTAB detergent molecules that were initially placed at the interface between the DNA and lipid bilayer did not show a strong affinity to the DNA (Figure S16) but quickly diffused into the surrounding water or into the lipid bilayer. This suggests that while DTAB facilitates the lipid reconstitution from mixed micelles in experiments, it is not an important factor for the stabilization of the interface between DNA and lipids once DLNs have formed.

Finally, we simulated the system with only seven instead of 21 PEG chains in the dsDNA circle which did not form DLNs in experiments. Likewise, the simulated DLN appeared less stable as the DNA slipped over the edge of the lipid bilayer (Figure 3H), although the PEG molecules remained associated with the lipid bilayer, with each PEG molecule occluding roughly twice as much lipid surface area per molecule as in the 1.5 PEG/turn system (Figure 3I). This further demonstrates the high affinity of PEG to the lipid edge, but the number of PEGs appears insufficient to stably hold the DNA ring in place, consistent with the experimental observation that stable nanodiscs could not be formed when only seven PEG-37 molecules instead of 21 were attached per DNA circle. The bilayer thickness, curvature, and the number of lipids observed along the bilayer edge were similar to the unsupported bilayer (Figure S15).

### Incorporation of Transmembrane Peptide

Bilayer mimetics are often used for the biophysical characterization of membrane proteins^67^ or for drug delivery of hydrophobic molecules.^68,69^ As proof of principle for such future applications, we incorporated the hydrophobic transmembrane domain (TMD) of synaptobrevin, a protein that is found in the membrane of synaptic vesicles and is involved in the formation of the SNARE complex.^70^ For this, we modified the C-terminus of the TMD peptide with a biotin and bound it to a streptavidin-modified quantum dot (QD). The QD facilitates ultracentrifugation analysis due to its high density, its red fluorescence emission (λ_em_ = 655 nm) and produces high-contrast features in TEM images. All components including DTAB-solubilized lipids, peptides, PEG-DLNs, and streptavidin-QDs were mixed followed by detergent removal (Figure 4A). The complexes were characterized by isopycnic centrifugation as described above. The resulting QD-modified DLNs produced a band with both green fluorescence from the Tf-PC lipid and red fluorescence from the QDs and shifted from F6 to F7 due to the high density from the added QDs (Figure 4B). TEM images of fraction 7 (Figure 4C) clearly show high contrast QDs bound to the DLNs. The outer diameters of the DLNs were 18.2 ± 1.9 nm in good agreement with the theoretical value of 18 nm for a 147 bp circle. Without PEG modifications or the biotinylated TMD peptide, QDs did not colocalize with the dsDNA rings (Figure S17), proving the incorporation of the TMD peptide in the DLNs.

**Figure 4:**
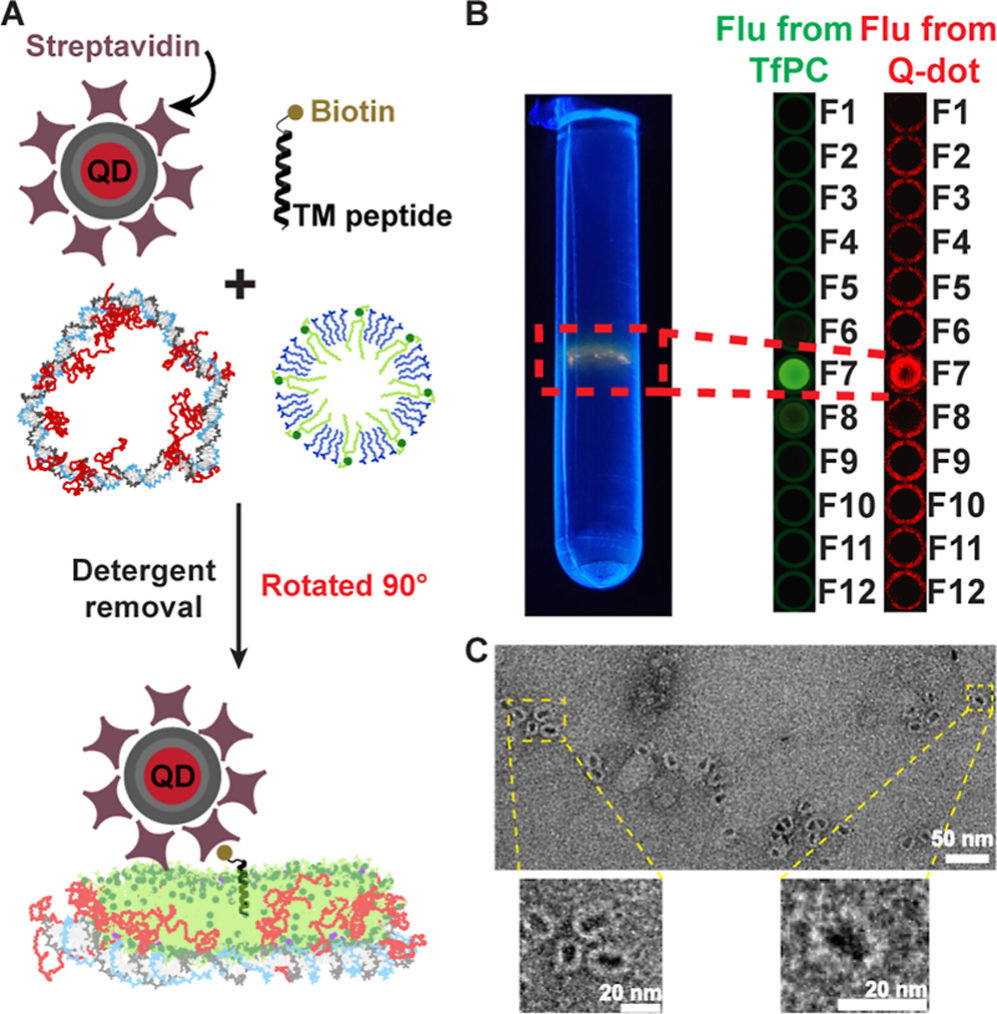
Incorporation of a transmembrane (TM) peptide into DLNs. (A) Incorporation of a biotin-modified transmembrane peptide in the lipid bilayer where biotin binds to a streptavidin-modified quantum dot (QD). (B) Isopycnic centrifugation of the DLN with the peptide and QD showing colocalized signals from fluorescent lipid and the streptavidin QDs indicating incorporation of the peptide in the DLN. (C) TEM image of DLN with a bound QD. Scale bar: 50 nm. Scale bar for zoomed-in images: 20 nm.

## Conclusion and Outlook

In this study, we developed a poly­(ethylene glycol)-modified DNA-based membrane mimetic to envelop lipid bilayers exploiting the favorable interactions between amphiphilic PEG chains and lipids. The conjugation of amphiphilic PEG chains of various lengths to multiple positions had little impact on the *T*_m_ of DNA to its template, but a minimum of three PEG 37 per oligonucleotide was necessary for DLN formation. Both reducing the length of PEG modifications and/or reducing the number of modifications significantly impacted the efficiency of lipid incorporation within the minicircles, while much longer PEG molecules inhibited incorporation of lipids. The optimized DNA circles contained 1.5 PEG 37 chains per helical turn and in ultracentrifugation experiments were quantitatively filled with lipids to form PEG-DLNs. Our atomistic MD simulations show qualitatively the same trends as experiments. They reveal that the PEG-minicircles stably hold lipids, but the DNA asymmetrically slipped toward the polar lipid head groups. The methylene groups of PEG chains interacted with the hydrophobic tails of the lipid bilayer, occluding a significant fraction of the solvent-exposed lipid tail surface area and mitigating the number of lipids that would roll over the edges of the bilayer. Additionally, the simulations reveal a flipping of positively charged lipids to the leaflet that is closest to the DNA circle and an enrichment of these positive lipids at the edge of the bilayer where they interact with the negatively charged phosphate backbone.

As a proof-of-concept, we incorporated the transmembrane domain of synaptobrevin that were bound to large streptavidin-modified QDs into our PEG-DLNs suggesting that large membrane proteins could also be incorporated.

In future work, we will develop this system further. The surprising slipping of the DNA to one of the leaflets exposes more of the lipid acyl chains than we expected and might be a reason why longer lipids such as DOPC and POPC are not compatible with our system, yet. We will therefore connect two DNA rings into a two-layer design or produce larger DNA origami rims. This might also allow us to easily synthesize much larger, yet fusion-stable nanodiscs. Furthermore, conjugation of established nanodisc-forming amphiphilic polymers such as SMA-based systems^13^ or peptides^10–12^ might enable a nanodisc synthesis without cationic lipids and detergents that structural biologists usually try to avoid. Potentially, modifications with membrane-solubilizing polymers will even enable a selective excision of membrane proteins from intact cellular membranes, which would circumvent the use of detergents altogether. The relatively quick flipping of lipids that was observed in simulations might, however, equilibrate the lipid asymmetry of natural membranes. We envision that DLNs will enable new applications in structural biology, biophysics, and biomedicine due to the modularity of the design and the possibility to extend the design with additional, site-specific functionalizations.

## Experimental Methods

### 1 Experimental Section

#### 1.1 Materials

All amine-modified DNA oligonucleotides were purchased from Integrated DNA technologies in the desalted form and were suspended in deionized water to form a 1 mM solution. They were purified in-house with reverse-phase high performance liquid chromatography (RP-HPLC) from Agilent using a Restek Viva 5 μm C4 column (cat. no. 9512525). The oligonucleotides were then dried using a vacuum centrifuge (Eppendorf) followed by resuspension in water to form a 1 mM solution. The yield of the purified oligonucleotides varied between 50 and 70%. PEG-3-NHS (cat. no. BP-20980), PEG-13-NHS (cat. no. BP-22584) and PEG-37-NHS esters (cat. no. BP-22248) were obtained from Broadpharm. DMF (cat. no. 043465) and sodium bicarbonate (cat. no. 470302-440) were obtained from Alfa Aesar and Ward’s science, respectively. 10× PBS (cat. no. J75889-AE) was purchased from Thermo Scientific. 1,2-Dimyristoyl-*sn*-glycero-3-phosphocholine (DMPC, cat. no. 850345),1,2-dimyristoyl-3-trimethylammonium-propane (chloride salt) (DMTAP, cat. no. 890860) and 1-palmitoyl-2-(dipyrrometheneboron difluoride)­undecanoyl-*sn*-glycero-3-phosphocholine (Topfluor (Tf) PC, cat. no. 810281) were purchased in the powder form from Avanti Research. Dodecyltrimethylammonium bromide (DTAB, cat. no. D5047) was purchased from Sigma-Aldrich. Optiprep density gradient medium (cat. no. AB286850) was obtained from Abcam. 96-well nontreated, sterilized plates were purchased from VWR (cat. no. 10861-562). QD 655 streptavidin conjugate (cat. no. Q10123MP) was obtained from Thermo Fisher Scientific. Chloroform (cat. no. CX1054-6) and methanol (cat. no. MX0488-1) were purchased from Millipore Sigma. Zymo-Spin IICR columns (cat. no. C1078), Oligo binding buffer (cat. no. D4060-1-40) and DNA wash buffer (cat. no. D4003-2-48) were purchased from Zymo research.

20% denaturing polyacrylamide gels were hand cast using 8 M Urea (VWR, cat. no. 0568), 10× TBE prepared in-house, 40% acrylamide-bis­(acrylamide) solution (19:1) from Thermo Scientific (cat. no. J60909.K2), 10% ammonium persulfate from VWR (cat. no. M133) and TEMED from Bio Rad (cat. no. 161-0801). GeneRuler Ultra Low Range DNA ladder was purchased from Thermo Fisher Scientific (cat. no. SM1211).

5% native gels with 5 mM MgCl_2_ were hand cast using 40% acrylamide-bis­(acrylamide) solution (29:1) from VWR (cat. no. 0311) and MgCl_2_ hexahydrate (VWR, cat. no. BDH9244). All gels were run in 1× TBE (100 mM TRIS, 100 mM Boric Acid, 2 mM EDTA, pH 8.3). Tris­(hydroxymethyl)­amino methane was purchased from Sigma-Aldrich (cat. no. 252859), Boric acid (cat. no. BDH9222) and disodium salt of EDTA were purchased from VWR (cat. no. BDH4616). SYBR gold (cat. no. S11494) and SYBR green (cat. no. S7563) DNA gel stains were purchased from Invitrogen by Thermo Fisher Scientific. HEPES (cat. no. J848) was purchased from VWR, MgSO_4_·7H_2_O (cat. no. M2773) and Na_2_SO_4_ (cat. no. 71959) were purchased from Sigma-Aldrich. 6× native DNA loading dye (cat. no. B7024S) and 100 bp DNA ladder (cat. no. N3231) were purchased from New England Biolabs. Uranyl formate hydrate was purchased from Electron Microscopy Sciences (cat. no. 22450).

#### 1.2 Experimental Details

##### PEGylation of Amine-DNA

5 μL of a 1 mM solution of HPLC-purified 3-amine oligonucleotides were mixed with 1.625 μL of 10× PBS pH 7.4 and 1.625 μL of 1 M NaHCO_3_ in a DNA-low bind microcentrifuge tube. This step causes deprotonation of the amine group making it suitable for nucleophilic attack of the lone pair at the carbonyl group of the NHS ester. To this solution, 8.04 μL of PEG-37 NHS ester (30-fold molar excess per amine) dissolved in anhydrous, amine-free DMF to a concentration of 100 mg/mL were added and mixed well. This reaction mixture with a total volume of ∼16.3 μL was incubated in a thermomixer at 25 °C, 1000 rpm for 12 h.

Postreaction, the sample was diluted with water to 100 μL and purified by RP-HPLC. The respective peaks were collected, and solvent was evaporated in a vacuum centrifuge and pellet was redissolved to make a 100 μM solution. The yields of the purified product were measured by UV absorbance and were about 80–90%.

Conjugation of the 1-amine oligonucleotide to PEG-37-NHS was carried out in a similar manner but the volume of NHS ester was proportionately lowered to 2.68 μL. Conjugations of 1- and 3-amine oligonucleotides to PEG-3 and PEG-13 NHS esters were also performed simultaneously according to the protocol described above.

Sequences of amine oligonucleotides using the modifier codes from IDT DNA:

1-amine: ACACTTTTTTTCACACTTTTTTC/3AmMO/

3-amine: /5AmMC12/ACACTTTTT/iAmMC6T/CACA­CTTT­TTTC/​3AmMO/

##### Denaturing Polyacrylamide Gel Electrophoresis

20% denaturing urea polyacrylamide gels were cast using 5 mL of 40% acrylamide-bis­(acrylamide) (19:1), 1 mL of 10× TBE, 4.8 g of urea, 100 μL of 10% APS, 10 μL of TEMED and water to make up the total volume to 10 mL. Gels were polymerized for 30–60 min before samples were loaded. 7.5 μL of 20 pmol of starting amine-DNA and the PEGylated product were each mixed with 2.5 μL of 4× denaturing loading dye (100% formamide, 10 mM NaOH, 0.01% bromophenol blue, 0.02% xylene cyanol) and loaded in the sample wells. 1 μL of 0.01 μg/mL of the GeneRuler ultralow range DNA ladder was also mixed with the denaturing loading dye and applied to sample wells as a reference. Electrophoresis was performed at 200 V for 65 min at 65 °C.

##### Formation of Single-Stranded Circular Scaffold and Double-Stranded Minicircles

Single-stranded circles were synthesized according to a previously published protocol.^14^ Briefly, a 147 nt linear oligonucleotide was phosphorylated at the 5′ end using T4 phosphonucleotide kinase (PNK) and the ends were splint ligated using T4 DNA ligase. Uncircularized strands and splints were removed by digestion with Exonuclease I and III. Pure circular product was purified by affinity columns (Zymo research) followed by yield estimation using absorbance measurements.

The sequence of single-stranded 147-mer DNA was 5Phos/(TGTG­AAAA­AAGT­GTGA­AAAAG)_7_.

Double-stranded (ds) minicircles were formed by annealing 500 pmol of the circular scaffold with 12.5 nmol of the complementary unmodified or PEGylated oligonucleotides in 5 mM MgCl_2_ by heating to 80 °C and slowly cooling them to 25 °C over an hour. The ds-circles were purified with 50 kDa molecular weight cutoff (MWCO) filters in a buffer containing 50 mM HEPES, 3 mM MgSO_4_ and 100 mM Na_2_SO_4_. The sample was washed 5–7 times with 500 μL of buffer and spun at 10,000 rcf for 5 min. Finally, the filter was inverted and the sample was retrieved by spinning at 10,000 rcf for 3 min. Yields were determined by measuring absorbance at 260 nm and the ds-circles were analyzed by native polyacrylamide gel electrophoresis (PAGE) or AFM.

##### Native Polyacrylamide Gel Electrophoresis

5% native polyacrylamide gels were cast using 1.25 mL of 40% acrylamide-bis­(acrylamide) (29:1), 1 mL of 10× TBE, 250 μL of 200 mM MgCl_2_, 100 μL of 10% APS, 10 μL of TEMED and 7.39 mL of water to make up the total volume to 10 mL. Native-SDS gels consisted of an additional 100 μL of 5% SDS in them. Gels were allowed to polymerize for 30–60 min before samples were loaded. 10 μL of 0.5 pmol of single-stranded circular scaffold were mixed with 2 μL of commercial 6× native loading dye (2.5% Ficoll-400, 10 mM EDTA, 3.3 mM Tris-HCl, 0.08% SDS, 0.02% Dye 1, 0.001% Dye 2, pH 8.0 at 25 °C) was loaded in the sample wells. For native-SDS gels, 10 μL of fractions after ultracentrifugation were mixed with 1.1 μL of 10× native-SDS dye prepared in-house (100 mM Tris-HCl, 50% glycerol, 0.5% SDS, pH 8.0 at 25 °C) and loaded. Since SYBR gold stains dsDNA better than ss DNA, only 0.25 pmol of ds-rings were loaded into gels. 1 μL of 50 μg/mL of the 100 bp DNA ladder was also mixed with the native/native-SDS loading dye and applied to sample wells as a reference. Electrophoresis was performed at 100 V for 65 min at 4 °C in a running buffer containing 1× TBE, 5 mM MgCl_2_ for native gels and an additional 0.05% SDS for native-SDS gels.

##### Denaturing and Native PAGE Staining and Imaging

When electrophoresis was complete, the gel was stained in 1× SYBR gold in 1× TBE supplemented with 5% ethanol to prevent the stain from sticking to the plastic staining tray. Gels were then imaged on a GE Typhoon FLE 9500 gel scanner using a 473 nm excitation laser and 510 nm long pass emission filter. The photomultiplier tube gain was set to 500 and the pixel size was 50 μm. Images were analyzed by the Fiji ImageJ image analysis software.

##### Atomic Force Microscopy

20 μL of a 0.01% polyornithine (Sigma-Aldrich, cat. no. P3655) solution were added onto freshly cleaved mica and incubated for 2 min. The mica surface was then washed with ∼10 mL of Milli-Q and dried with a stream of N_2_ gas. 10 μL of unmodified or PEGylated ds-rings (20 nM) were deposited onto the mica (Ted Pella, 9.9 mm diameter) and incubated for 3–5 min. The solution was wicked away and the surface was washed twice with 1 mL of Milli-Q water after which 60 μL of water were added on to the sample surface. 20 μL of water were also added onto the ScanAsyst Fluid+ tip (Bruker, cat. no. p-3728) and imaging was performed in liquid using the peak force tapping mode in a highspeed atomic force microscope (Bruker, Dimension Fastscan bio). Images were processed with the Nanoscope analysis software.

##### Transmission Electron Microscopy

400-mesh carbon-only grids (Electron Microscopy Sciences, cat no. CF400-Cu-UL) were glow-discharged for 30 s at 15 mA negative polarity using a PELCO easiGlow glow discharge system to render the surface hydrophilic and facilitate sample deposition in the subsequent steps. For optimum results, glow-discharged grids were used within 15 min of treatment. 5 μL of the purified ds-PEG-minicircles or PEG-DLNs or QD-peptide-PEG-DLNs were applied to treated grids and incubated for 5 min. Meanwhile, a fresh 1% uranyl formate solution in water was prepared. To this, 1.25 μL of freshly prepared 1 M NaOH were added, mixed well for 5 min in a thermomixer at 750 rpm, and centrifuged for 30 s using a tabletop microcentrifuge. We observed that preparation of fresh uranyl stain showed dramatic improvement in results compared to uranyl stains that were stored at −20 °C for extended periods of time. After the sample was incubated for 5 min, the excess sample was wicked away, and 10 μL of freshly activated uranyl formate were applied to the grid and incubated for 30 s before the excess was wicked away. The grids were then air-dried and imaged using a Tecnai F20 transmission electron microscope operated at 200 kV. Images were analyzed with the Fiji ImageJ analysis software.

##### *T*_m_ Analysis

A 21-mer DNA which is the exact complement of the PEGylated oligonucleotide was designed (sequence: GAAA­AAGT­GTGA­AAAA­AGTGT). 1 μL of the 21-mer DNA (200 μM) was mixed with 1 μL (5-fold molar excess) of the complementary unmodified or PEGylated oligonucleotide (1 mM) in 1× TE buffer with 5 mM MgCl_2_. To this 3 μL of 10× SYBR green (such that final concentration was 1×) were added to record real-time change in fluorescence upon DNA hybridization. The total reaction volume was 30 μL. Additionally, samples were also layered with oil to prevent evaporation. Samples were placed in a real-time magnetic induction cycler (Biomolecular Systems) and heated to 85 °C for 2 min through magnetic induction followed by cooling to 40 °C over an hour using fan-forced air. Fluorescence from the DNA-binding dye increased as the temperature decreased due to formation of dsDNA and was recorded with high-sensitivity photodiodes for each dedicated excitation/emission channel. The micPCR software was used to take the first derivative of the change in fluorescence with respect to temperature (d*F*/d*T*) to obtain the *T*_m_ of the duplex.

##### Lipid Film Preparation

Powder stocks of DMPC, DMTAP and TfPC were dissolved in 2:1 chloroform/methanol to make a stock concentration of 25 mg/mL for DMPC, DMTAP and 1 mg/mL for TfPC respectively. Stock solutions of lipids were used immediately and are not recommended for long-term storage. 12.06 μL of DMPC, 1.18 μL of DMTAP and 5 μL of TfPC in the ratio of 89:10:1 respectively, were mixed in a clean glass test tube. The solvent was evaporated using a stream of N_2_ gas while the tube was placed in hot water to ensure all lipids remained above their phase transition temperature as they dry. The dried lipid films were placed in a vacuum desiccator overnight. Finally, the tubes were purged with N_2_, sealed and stored at −20 °C until further use.

##### Nanodisc Preparation

Lipids were dissolved to a final concentration of 2 mM in 1× buffer H (50 mM HEPES, 3 mM MgSO_4_ and 100 mM Na_2_SO_4_) with 60 mM DTAB and sonicated for 10 min to form mixed micelles. 8.53 μL (25 pmol) of DNA-PEG-minicircles in 1× buffer H were mixed with 5.62 μL (11.25 μmol) of lipids at a molar ratio of 1:450 (DNA/lipid). 13.23 μL of 100 mM DTAB were added to adjust the final concentration to 20 mM to ensure it is above the critical micelle concentration (cmc) of DTAB which is ∼15 mM. The total volume was 83 μL and was adjusted by addition of 1× buffer H to achieve a final DNA and lipid concentration of 300 nM and 135 μM respectively. Samples were incubated at 30 °C (above the phase transition temperature of DMPC which is 25 °C) overnight followed by detergent removal using Pierce detergent removal spin columns (cat. no. 87777, Thermo Scientific) according to the manufacturer’s protocol. Briefly, the spin columns were spun down at 1500*g* for 1 min to remove storage buffer. Columns were equilibrated by three additions of 400 μL of 1× buffer H followed by centrifugation at 1500*g* for 1 min. Next, the sample was added, incubated for 2 min and centrifuged at 1500*g* for 2 min to retrieve the detergent-free PEG-DLNs in 83 μL of 1× buffer H.

##### Density-Gradient Ultracentrifugation

Optiprep density gradient medium (60% iodixanol) was used to prepare different gradients ranging from 2% to 26% in 1× buffer H (Table 1).

**Table 1:** Preparation of Density Gradients for Ultracentrifugation Experiments

percentage	Optiprep medium (μL)	10× buffer H (μL)	water (μL)
2	16	48	416
6	48	48	384
10	80	48	352
14	112	48	320
18	144	48	288
22	176	48	256
26	208	48	224

83 μL of the detergent-free PEG-DLNs were mixed with 83 μL of 55% iodixanol to prepare a 27% iodixanol solution. 0.8 μL open-top thin wall ultracentrifuge tubes (Beckman Coulter Life Sciences, cat. no. 344090) were used for this experiment. 166 μL of the sample + iodixanol (27% solution) was added to the tube followed by layering with 70 μL of 26%, 22%, 18%, 14%, 10%, 6% and 2% solutions, respectively. The tube was placed with its adapters (Beckman Coulter Life Sciences, cat. no. 356860) in a SW55Ti swinging bucket rotor (Beckman Coulter Life Sciences, cat. no. 342194). Samples were spun at 45,000 rpm (∼245,000*g*) for 6 h at RT after which the contents were fractionated into 96-well plates, followed by analysis of lipid signals using fluorescence and DNA by native-SDS PAGE respectively. Fractionated samples were imaged on a GE Typhoon FLE 9500 gel scanner using a 473 nm excitation laser and 510 nm long pass emission filter (ex/em maxima for TfPC lipid). The photomultiplier tube gain was set to 500 and the pixel size was 50 μm. Fluorescence signals were analyzed by the Fiji ImageJ image analysis software.

##### Transmembrane Peptide Incorporation

The transmembrane (TM) domain of synaptobrevin was modified to contain a biotin at the C-terminal end. The 23-amino acid peptide contains no charged amino acids (IILGVISAIILIIIIVYFSTGSS-Biotin, average molecular weight = 2674.4 g mol^–1^). The C-terminal “GSS” sequence is an α helix breaker and was added to provide flexibility. The peptide was solubilized to a final concentration of 50 μM in an aqueous 100 μM DTAB solution. 8.53 μL (25 pmol) of DNA-PEG-minicircles in 1× buffer H were mixed with 5.62 μL (11.25 μmol) of lipids and 1 μL (50 pmol) of peptide solution. This results in a 1:450 ratio of DNA/lipid and 1:2 ratio of DNA/peptide. To this solution, 13.23 μL of 100 mM DTAB were added to adjust the final concentration to 20 mM to ensure it is above the critical micelle concentration (cmc) of DTAB which is ∼15 mM. Then, 2 μL of 1 μM QD-streptavidin conjugate were added and mixed well. An excess of the QD-streptavidin could not be added due to low concentration of the commercially available conjugate. The total volume was 83 μL and was adjusted by addition of 1× buffer H to achieve a final concentration of 300 nM DNA and 135 μM of lipids. Samples were incubated at 30 °C (above the phase transition temperature of DMPC which is 25 °C) overnight followed by detergent removal using Pierce detergent removal spin columns (cat. no. 87777, Thermo Scientific) according to the manufacturer’s protocol to retrieve detergent-free PEG-DLNs with a TM peptide bound to QD-streptavidin.

After ultracentrifugation, samples were fractionated and imaged on a GE Typhoon FLE 9500 gel scanner using a 473 nm excitation laser with a 510 nm long pass emission filter (ex/em maxima for TfPC lipid) and a 635 nm excitation laser with a red long pass emission filter (ex/em maxima for the QD conjugate). The photomultiplier tube gain was set to 500 and the pixel size was 50 μm. Fluorescence signals were analyzed by the Fiji ImageJ image analysis software.

##### Synthesis of Biotinylated Transmembrane Peptide

The peptide NH_2_-IILGVISA­IILIIIIV­YFSTGSS-Biotin (MW = 2632.45 g/mol) was synthesized using microwave-assisted solid-phase peptide synthesis (SPPS) on a CEM Liberty Blue 2.0 instrument employing Fmoc-chemistry. Biotin Nova Tag resin was used as the solid support, with amino acid activation carried out using *N*,*N*′-diisopropylcarbodiimide and Oxyma in DMF, and piperidine as the base. Resin cleavage and side chain deprotection were performed using a mixture of trifluoroacetic acid (87.5% v/v), phenol (5% v/v), triisopropyl silane (2.5% v/v), and water (5% v/v) for 2.5 h at room temperature (21 °C). The crude peptide was then precipitated in diethyl ether and collected by vacuum filtration.

### 2 Atomistic Molecular Dynamics Simulations

#### 2.1 General Simulation Methods

All-atom simulations were performed using NAMD 3.0.1 ^46^ and employed the CHARMM36 ^47,48^ force field with CUFIX^49–51^ corrections, TIP3P^52^ water model, permanent magnesium hexahydrates,^50^ and periodic boundary conditions. Hydrogen mass repartitioning^53^ and the SHAKE^54^ and SETTLE^55^ hydrogen constraint algorithms were used, enabling a 4 fs integration time step. van der Waals and short-range electrostatic forces were evaluated using an 8–12 Å smooth cutoff scheme. Long-range electrostatic interactions were computed using the particle-mesh Ewald method^56^ with a 1 Å grid spacing. The temperature was held at 310 K by a Langevin thermostat applied to non-hydrogen atoms with 1 ps^–1^ damping coefficient. The pressure was maintained at 1 atm by a Nosé–Hoover Langevin piston barostat^57,58^ with a 2000 fs period and 1000 fs decay. When present, base-pairing hydrogen bonds were reinforced using additional harmonic bonds with 2.85 Å rest length and 100 kcal mol^–1^ Å^–2^ spring constant. The configuration of a system was written to a trajectory file every 10 ps of simulation.

#### 2.2 System Assembly and Simulation of DLN and Bare Lipid Systems

An idealized model of a DNA ring composed of 147-bp of DNA with sequence (GTGA­AAAA­AGTG­TGAA­AAAGT)_7_ and containing seven evenly distributed nicks was prepared using a custom script that employed a brief restrained mrDNA^59^ simulation to ensure the adenine nucleotides would face the outside of the ring. VMD^60^ and the psfgen program were used to combine a 79 Å radius patch of 90% DMPC, 9% DMTAP and 1% DMPE (564 total lipids) cut to fit, without steric clashes, inside the DNA ring functionalized by 3-amine linkers per to PEG per 21 nt ssDNA fragment (described in the Experimental Details section), and 80 DTAB molecules arranged near the DNA. The lipid bilayer described above was excised from an idealized patch generated by CHARMM-GUI.^61,62^ The entire system was submerged in a 21 × 21 × 8.5 nm^3^ box of 100 mM Na_2_SO_4_ and 6 mM MgCl_2_ electrolyte, while the system was made neutral by adding ∼45% of the solute charge in additional Mg^2+^ ions and removing Cl^–^ and SO_4_^2–^ corresponding to ∼5% and of the 50% of the excess charge, respectively.

After assembly, conjugate gradient minimization was applied to the system for 1000 steps, followed by equilibration with harmonic potentials restraining non-hydrogen lipid atoms to their initial coordinates, excluding DTAB using a 1 kcal mol^–1^ Å^–2^ spring constant that was halved every 16 ns for seven cycles (112 ns total) after which the restraints were fully removed. Simultaneously, during the first 120 ns of equilibration (including lipid-restrained equilibration), an mrDNA-generated elastic network of harmonic restraints (*k*_spring_ = 1 kcal mol^–1^ Å^–2^; rest length from distance in idealized helical geometry) that preserve base pairing and stacking was applied to the DNA. After the initial 120 ns, all elastic network restraints were removed except for those restraining base pair hydrogen bonds, which were applied throughout the production simulation. After 378 ns of simulation, the DLN had developed a significant tilt with respect to the short axis of the system, raising the possibility that the tilted DLN would form direct contacts with its periodic image. To avoid that possibility, a colvars^63^ tilt angle variable was created that included every fourth lipid C2 atom, using the initial configuration as a reference. The tilt angle is calculated by decomposing the rotation that best aligns the C2 atoms into two rotations, a spin rotation about the *z*-axis of the system (initially normal to the bilayer), and a tilt rotation in the *xy*-plane. The collective variable was calculated every 100 steps and was used in a harmonic restraint that guided the cosine of the tilt angle from 0.9952 (∼5.6°) to 1 over a 10 ns period using a stiff 10,000 kcal mol^–1^ (∼3 kcal mol^–1^ degree^–2^) spring constant. The potential acting on the tilt angle applied a maximum torque of 373 pN nm during the simulation. While a substantial torque, the potential by design cannot apply any internal force or stress to the constituent atoms. Accordingly, it is unlikely that the potential affects the structural properties of the DLN.

A second DLN system with 0.5 PEG per DNA turn system was constructed as described above, except the functionalized DNA strands contained only the central PEG molecule. The simulations were performed as described above, except the restraints on the lipid bilayer and the DNA elastic network restraints were removed after 108 ns, and the colvars tilt angle restraint was applied from the start of the simulation. Finally, a complementary “bare lipid” system designed to provide a comparison without the DNA scaffold was constructed using identical protocols as described above, except that the functionalized DNA was not added to the system, and the charge of the system was neutralized by adding excess SO_4_^2–^. The simulation protocols were identical to the DLN system, except that the absence of DNA in the system ensured that no contacts could be made between periodic images of the bilayer, even without colvars tilt angle restraints.

#### 2.3 Analysis of MD Trajectories

Analysis of the simulations was performed with VMD and MDAnalysis [Bibr newref64],[Bibr newref65] using preprocessed trajectories where the coordinates of the system were transformed to place the center of mass of the lipid bilayer C2 atoms (representative of the headgroup) at the origin and align their principal axes with Cartesian axes, simplifying subsequent calculations. All time-averaged quantities were computed after the first 250 ns of simulation, including the period of restrained equilibration.

The diffusion coefficient of each lipid species was estimated by calculating the mean squared displacement (MSD) of the C2 atom each lipid in the center of the DLN (radius <4 nm) for strides ranging from 1 to 128 ns in powers of two. For the MSD, the displacements were projected into the plane of the bilayer. The diffusion coefficient was calculated as one-quarter of the slope of a linear regression applied to the MSD, consistent with diffusion in a two-dimensional space, obtaining 0.02, 0.14, and 1.14 Å^2^/ns for DMPC, DMTAP and DMPE, respectively. From these coefficients, a lipid can be expected to diffuse 8.4, 22, or 63 Å, respectively, during the 880 ns production simulation. Those distances are comparable to the approximate distance between lipids of each species—9.4, 30, and 89 Å, respectively.

The occluded fraction of solvent accessible surface area of the lipid tails was estimated using the “measure sasa” feature of VMD with a 2.0 Å probe radius to compute the accessible area of the lipid tails with and without considering PEG as part of the solvent. Subtracting the areas provides the lipid tail area occluded by the PEG and DTAB, and dividing by the area that treats PEG as part of the solvent provides the occluded fraction solvent accessible lipid tail area. The analysis of contacts was performed using the “measure contacts” feature of VMD with a 4 Å cutoff to consider two atoms in contact.

The fraction of DMTAP or DTAB in a given region was computed by counting the number of C2 atoms of the molecule of interest found within the region, and dividing by the total number of lipid (plus DTAB) C2 atoms found in the region. The regions were defined by bisecting the bilayer into two leaflets and according to proximity to the bilayer edge for a given configuration according to the following procedure. The measure volinterior feature of VMD with an isovalue of 0.01 and resolution of 6 to extract a molecular surface of all lipid atoms (excluding DTAB), marking voxels as inside or outside the surface. Subsequently, a distance from the edge was calculated for each molecule using distance projected into the plane of the bilayer of its C2 atom from the center of the nearest pixel that was, on average over the normal axis of a 2 nm thick slab centered on the bilayer and sharing its normal, outside the bilayer. Except where specified, characterization of bilayer properties described below involved analysis of only those lipids with C2 atom contained within a 4 nm radius cylinder centered on the bilayer with axis along the bilayer normal. For a given configuration, the thickness of the bilayer was assessed by computing the density profile along the bilayer normal in 0.5 Å bins and fitting a Gaussian distribution to the density for each leaflet. The area pear lipid headgroup was determined by counting the C2 atoms in the 4 nm radius cylinder. The curvature was estimated by performing a nonlinear least-squares fit of a sphere to the lipid headgroup coordinates of each leaflet with shift along the bilayer normal and curvature taken as independent variables; the result obtained for the two leaflets were averaged. The number of lipid headgroups at the edge of each system was computed as the number of lipid C2 atoms in the 1 nm thick slab centered along the bilayer and sharing the bilayer normal vector with no restriction on which lipids are considered for the analysis.

## Supplementary Material







## Notes

Movie S1 (MP4)
Movie S2 (MP4)
Additional electrophoresis data, HPLC chromatograms, MS data, *T*_m_ data, ultracentrifugation results and additional simulation analyses, movies of the simulation trajectories and descriptions of the movies (PDF)
