# Supplementary material for: DNA-Lipid Nanodiscs with a Polyethylene Glycol Interface
Source: J Am Chem Soc. 2026 May 4;148(18):19214–25. doi: 10.1021/jacs.6c03471 (PMC13185122; doi:10.1021/jacs.6c03471)
Supplement: Supplementary file 3 [file ja6c03471_si_003.pdf]

# *DNA-Lipid Nanodiscs with a Polyethylene Glycol Interface*

*Soumya Chandrasekhar[1,2], Christopher Maffeo[3,4], Sanjai Karanth[1], Rachel Bricker[1], Joy Kabuga[6], Diana Patricia Nunes Gonçalves[6], Aleksei Aksimentiev[3,4,5], Thorsten L Schmidt*[1,2]*

*[1]Department of Physics, Kent State University, Kent, OH, 44242, USA. [2]Advanced Materials and Liquid Crystal Institute, Kent State University, Kent, OH, 44242, USA. [3]Department of Physics, University of Illinois at Urbana Champaign, Urbana, IL, 61801, USA. [4]Beckman Institute for Advanced Science and Technology, University of Illinois at Urbana Champaign, Urbana, IL, 61801, USA. [5]Department of Bioengineering, University of Illinois at Urbana Champaign, Urbana, IL, 61801, USA. [6]Department of Chemistry, Kent State University, Kent, OH, 44242, USA.*

Email: tschmi21@kent.edu

# Supporting information

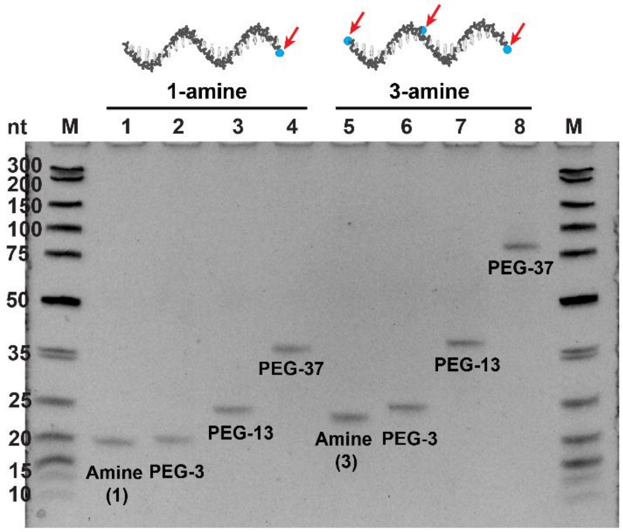


**Figure S1:** Denaturing gel electropherogram of PEGylated oligonucleotides. M: Ultra-low range DNA ladder. Lanes 1-4: 21-base oligonucleotide with one amine modification before and after reaction with PEG-3, PEG-13 and PEG-37 NHS esters respectively. Lanes 5-8: 21-base oligonucleotide containing three amine modifications before and after reaction with PEG-3, PEG-13 and PEG-37 NHS esters respectively.

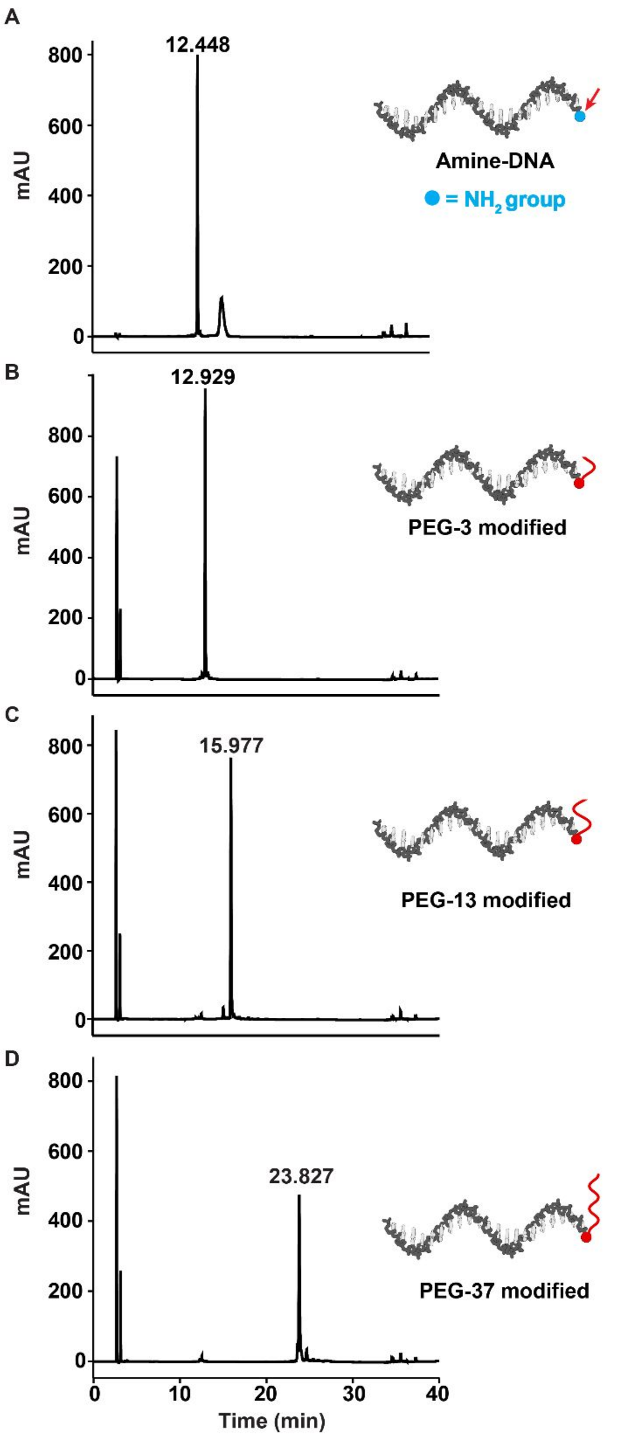


**Figure S2.** HPLC chromatograms of PEGylated oligonucleotides. (A) HPLC chromatogram of starting 1 amine oligonucleotide with retention time ~12 min. 1 amine oligonucleotide coupled to (B) PEG-3 NHS ester with a shift in retention time to ~13 min, (C) PEG-13 NHS ester with a shift in retention time to ~16 min, (D) PEG-37 NHS ester with a shift in retention time to ~24 min. Increasing shifts in retention time are attributed to the increasing hydrophobicity of the PEG chain lengths. Gradient: 3% to 30% acetonitrile in 30 min; 30% to 100% acetonitrile in the next 5 min; column wash with 100% acetonitrile for 3 min.

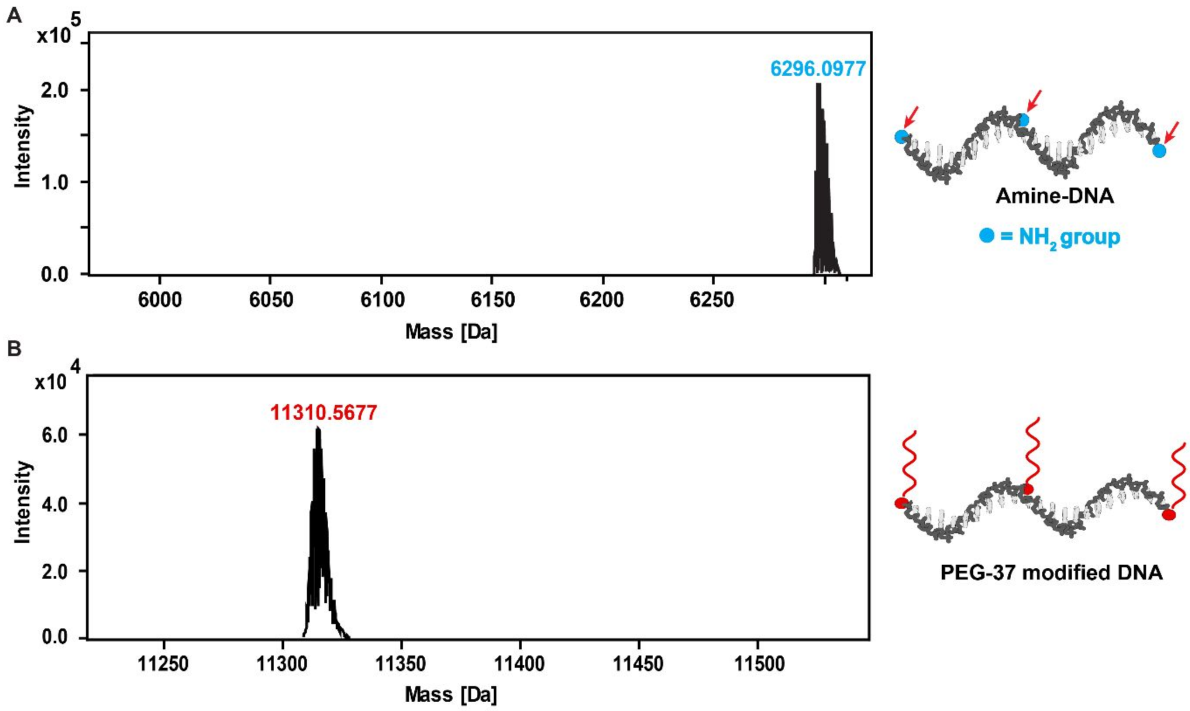


**Figure S3**. Mass spectra. Deconvoluted high-resolution mass spectra after HPLC-ESI-MS: (A) 3-amine oligonucleotide, expected average mass = 6298.4 $gmol^{-1}$, (B) 3 PEG-37-oligonucleotide conjugate, expected average mass = 11311.43 $gmol^{-1}$. Note that the peak pattern comes from the natural isotope distribution and only the highest peak is labeled for clarity.

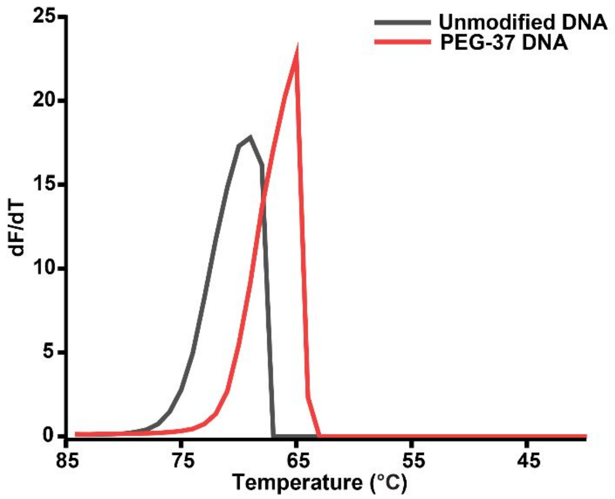


**Figure S4.** $T_m$ analysis for PEGylated DNA. Melting temperature ($T_m$) obtained by plotting first derivative of change in fluorescence with respect to temperature against change in temperature (dF/dT). Black: DNA without amine group ($T_m$ = 69 °C). Red: DNA with 3 PEG-37 modifications ($T_m$ = 65 °C).

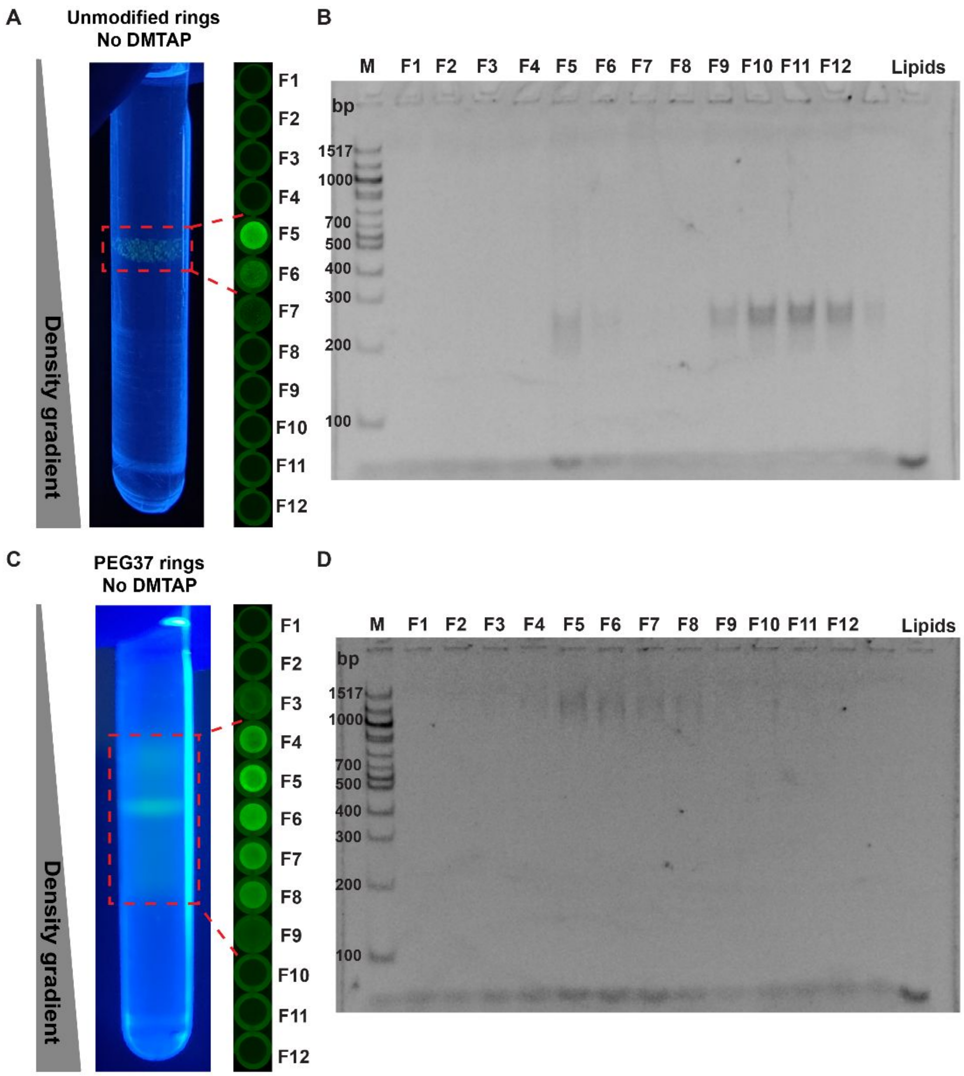


**Figure S5**. Reconstitution in the absence of cationic lipid. DLN synthesis attempts with ds-minicircles and DMPC, TfPC without any DMTAP. A-B: reconstitution with unmodified ds-minicircles, C-D: reconstitution with PEG-ds-minicircles. (A-B) Fractions 9-12 show most ds-minicircles do not colocalize with lipids except for F5-F6 which show colocalization due to the formation of an electrostatic aggregate. In C-D, ds-minicircles are observed in F4-F10 and colocalize with lipids. However, the fluorescence is distributed over several fractions and does not appear concentrated as in samples with DMTAP. See Figure S8 for additional experimental details.

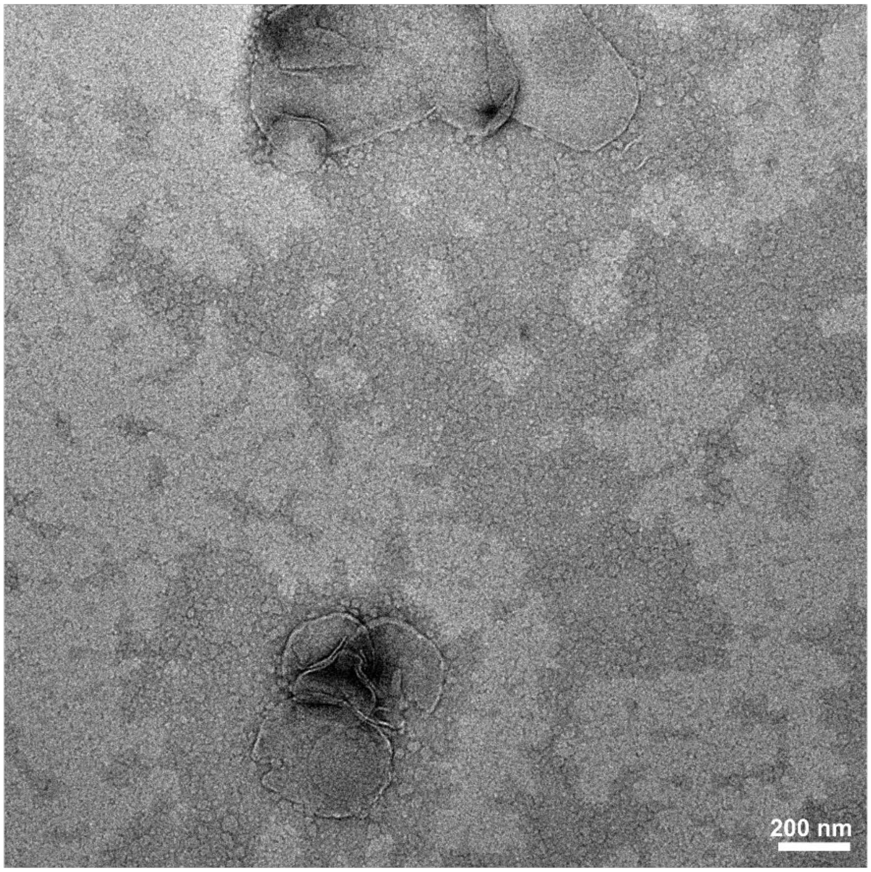


**Figure S6. Another TEM image of DLN sample after detergent removal.** In addition to DLNs, this area of the grid also shows liposomes that can be distinguished well from much smaller DLNs.

Note that a precise size determination of DLNs from these images is problematic for several reasons. Unpurified samples contain some DLNs with twice the circumference and diameter of the designed circle, as the splint ligation of the ss minicircle scaffold can form circular ss dimers as a side product. Although PAGE analysis of the ss circles after splint ligation shows that dimers account for only ~10% of total ss circular scaffolds, larger DLNs seem overrepresented in the images. We attribute this to better adhesion capabilities to TEM grids due to their higher surface area, and had already observed this effect previously.[1] Perhaps, neighboring DLNs also fuse during drying and uranyl staining of the sample, giving rise to larger nanodiscs.

We also tried to image purified nanodiscs after ultracentrifugation, but found them not to stick well enough, neither to plasma-treated nor to untreated grids. Moreover, they were much more diluted in the iodixanol fractions, and remaining iodixanol seems to weaken adsorption even further. A buffer exchange and concentration by ultrafiltration caused large losses, presumably due to adsorption of cationic lipids in the lipid formulation to the ultrafiltration membranes.

We had similar adsorption and imaging problems in our previous design, where DLNs where pushed around on a mica surface during AFM imaging.[1] We hypothesize that in this new PEGylated design, the entropic brush effect of the PEG molecules worsens the adhesion problems. We had also observed weaker adsorption of PEG-coated DNA origamis, which rearranged on TEM grids during drying.[2]

In summary, PEGylated DLNs could only be imaged well right after detergent removal and only when drying a thicker layer of solution than we would typically leave in the preparation of DNA origamis or other samples that adhere well to the grid surface.

Note that purified nanodisc images with a QD (Figure 4C) have better-defined edges and show mostly monomeric rings with exactly the right size, albeit at lower overall density. The better contrast and circularity could come from hydrophilic, streptavidin-modified QDs landing on the TEM grid with the QD down, preventing diffusion and fusion of rings and lipids during drying and staining of the sample.

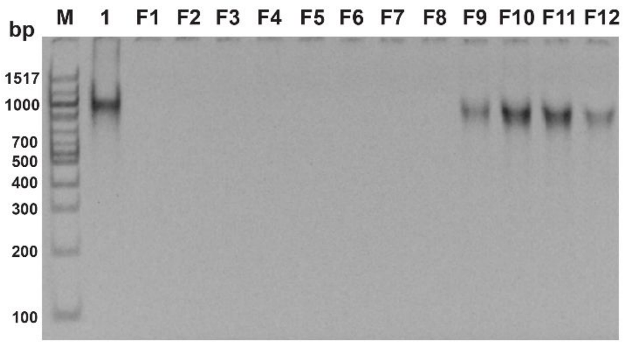


**Figure S7.** Gel electropherogram of a PAGE gel containing SDS and $MgCl_2$ of the fractions collected after isopycnic ultracentrifugation showing the ds-PEG-minicircles in the bottom fractions (F9-F12). M: 100 bp dsDNA ladder. Lane 1: ds-PEG-minicircle (control).

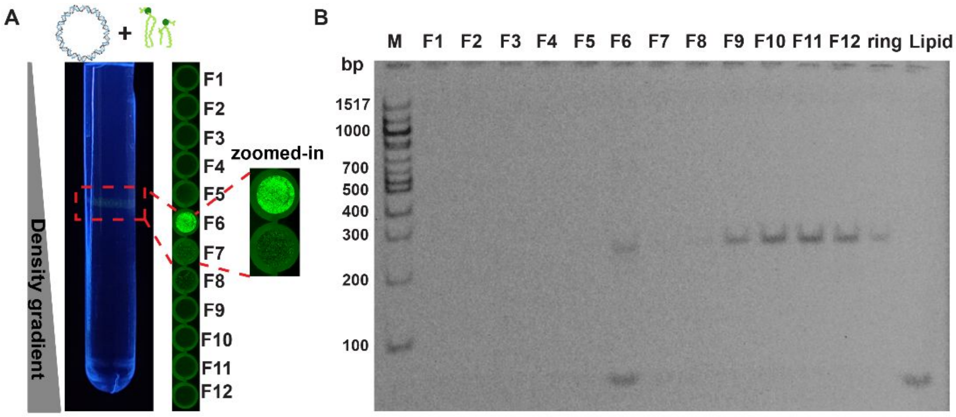


**Figure S8.** Control experiment without PEG modifications. Analysis of a DLNs experiment with unmodified ds-minicircles (no PEG modification). The lipid formulation contained 1% fluorescent lipid (top fluor-PC). (A) The photograph of the tube after ultracentrifugation was taken under UV illumination to visualize fluorescent lipids. The content of the ultracentrifugation tube (left) was fractionated into the wells of a 96-well plate and imaged in a fluorescence scanner (right), indicating lipids in the 6$^{th}$ and 7$^{th}$ fraction (F6-F7). Note that the lipids appear visually aggregated in the zoomed-in fluorescence scan, indicating that nanodiscs are unstable. (B) PAGE analysis of the fractions taken from (A). The gel contained SDS to solubilize lipids and $MgCl_2$ to stabilize dsDNA and stained with Sybr Gold to detect DNA (band comigrating with the 300 bp band of the ladder). The majority of the ds minicircles was found in fractions 9-12, not colocalized with the lipid signal (lower band in F6). The small fraction of the minicircles in F6 is possibly due to the formation of an electrostatic complex. M: 100 bp dsDNA ladder. Ring: Unmodified ds-minicircle (control); control lipids were solubilized in SDS and form mixed micelles migrating a little faster than the 100 bp marker lane.

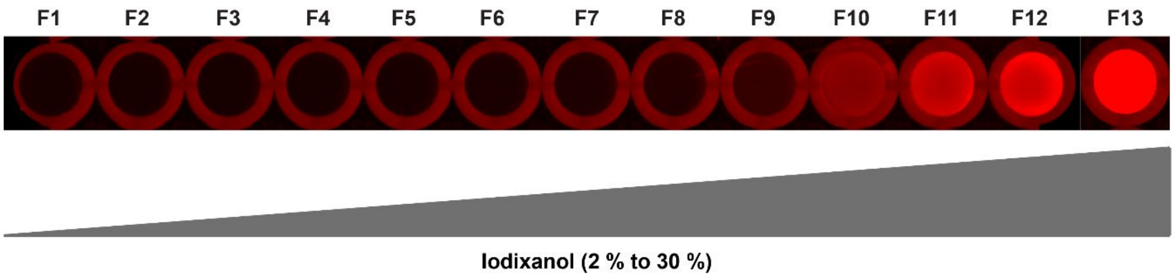


**Figure S9.** Non-specific interactions of Nile red (lipid stain) with iodixanol (density gradient medium). Fractions F1-F13 contain increasing amounts of iodixanol (in buffer) ranging from 2-30 %, fractionated after isopycnic ultracentrifugation. A constant amount of the Nile red was added to each well and imaged in a fluorescence scanner with a 532 nm excitation laser. This demonstrates that lipid bilayers cannot selectively be detected with Nile red due to the non-specific staining with iodixanol.

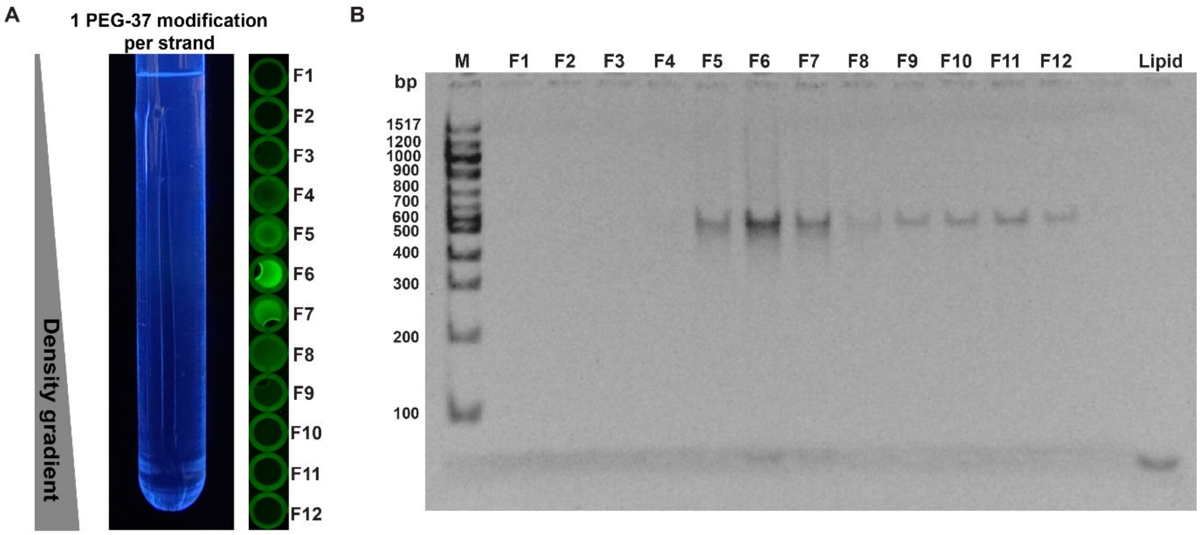


**Figure S10.** Reducing the number of PEG modifications. DLNs synthesis is inefficient with ds-PEG-minicircles containing only one PEG-37 modification per strand, or seven PEG-37 modifications per ds-ring instead of 21. (A) No sharp band is observed (A) and instead lipids are spread around fractions F5-F7, while ds minicircles appear in fractions F5-F12 (B). See Figure S8 for additional experimental details.

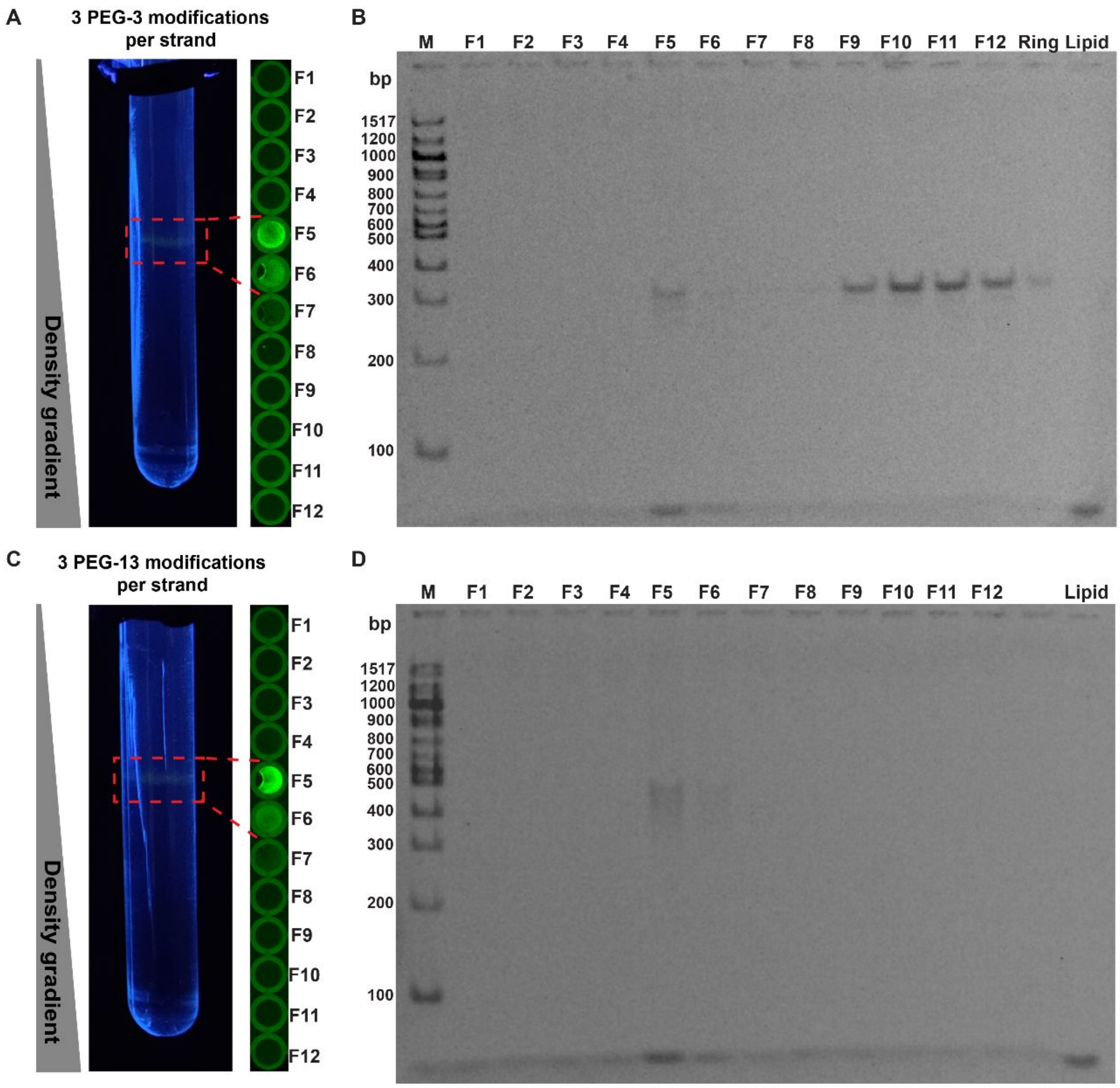


**Figure S11.** Shortening PEG chains. DLNs are not efficiently formed with ds-minicircles with shorter PEG modifications. ds-Minicircles contained 21 PEG-3 (A-B; three ethylene glycol repeats) or 21 PEG-13 modifications per ds-ring (C-D) instead of the optimized PEG-37 chain length. In both cases, lipids formed a fuzzy bands around F5-6, but only a fraction of DNA (upper band) colocalized with the lipids. The overall intensity is low indicating significant loss of sample in the detergent removal step. See Figure S8 for additional experimental details.

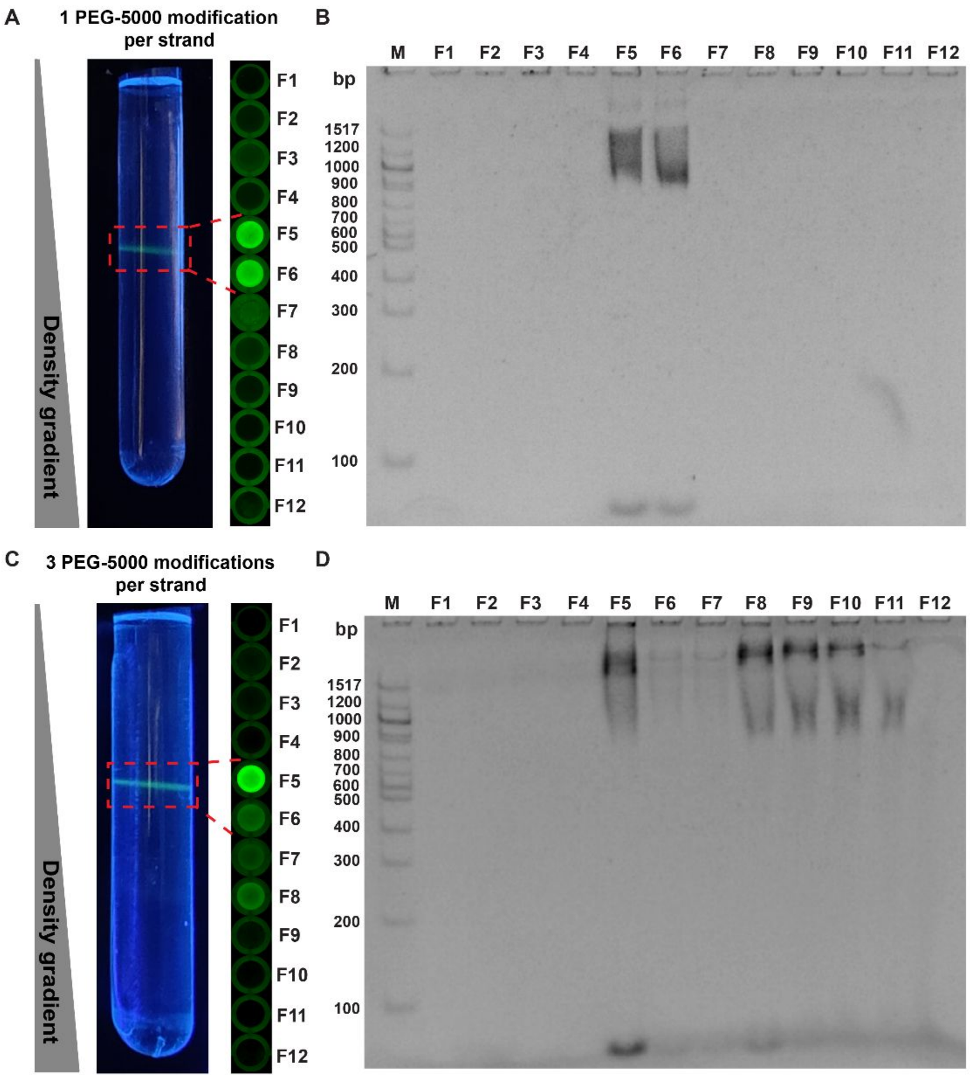


**Figure S12.** Increasing the PEG chain length. DLN synthesis attempts with ds-minicircles with longer PEG-5000 (~5 kDa) instead of PEG-37 (~1.7 kDa) modifications. A-B: one modification per strand or seven PEG chains per ds-minicircle, C-D: three modifications per strand or 21 per ds-minicircle. (A-B) Fractions 5-6 show most ds-minicircles colocalized with lipids indicating successful DLN formation. However, in C-D, some ds minicircles (F8-10) do not colocalize with lipids suggesting that extensive modification with long PEG chains hinders DLN formation. Note that unlike the atomically precise PEG-37, PEG-5000 is a polydisperse polymer and therefore DNA bands are more smeared out. See Figure S8 for additional experimental details.

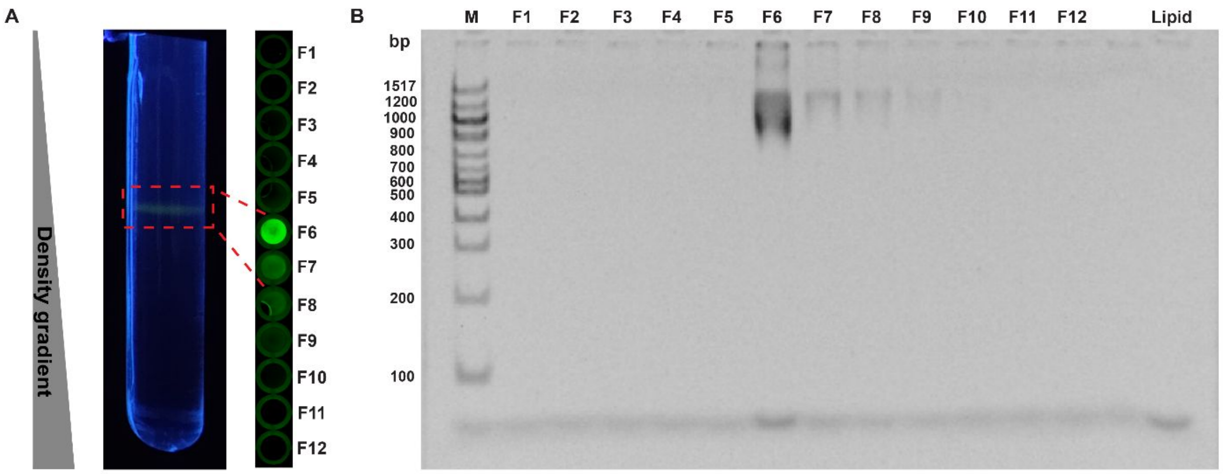


**Figure S13.** Increasing minicircle flexibility. In this experiment, were designed ds-PEG-minicircles with 21 PEG-37 modifications to have a two-nucleotide single-stranded gap between all seven oligonucleotides on ss scaffold ring. While lipids colocalized with the ds minicircles indicating successful DLN formation, the results did not show any improvement over the design without gaps and was therefore discontinued. See Figure S8 for additional experimental details.

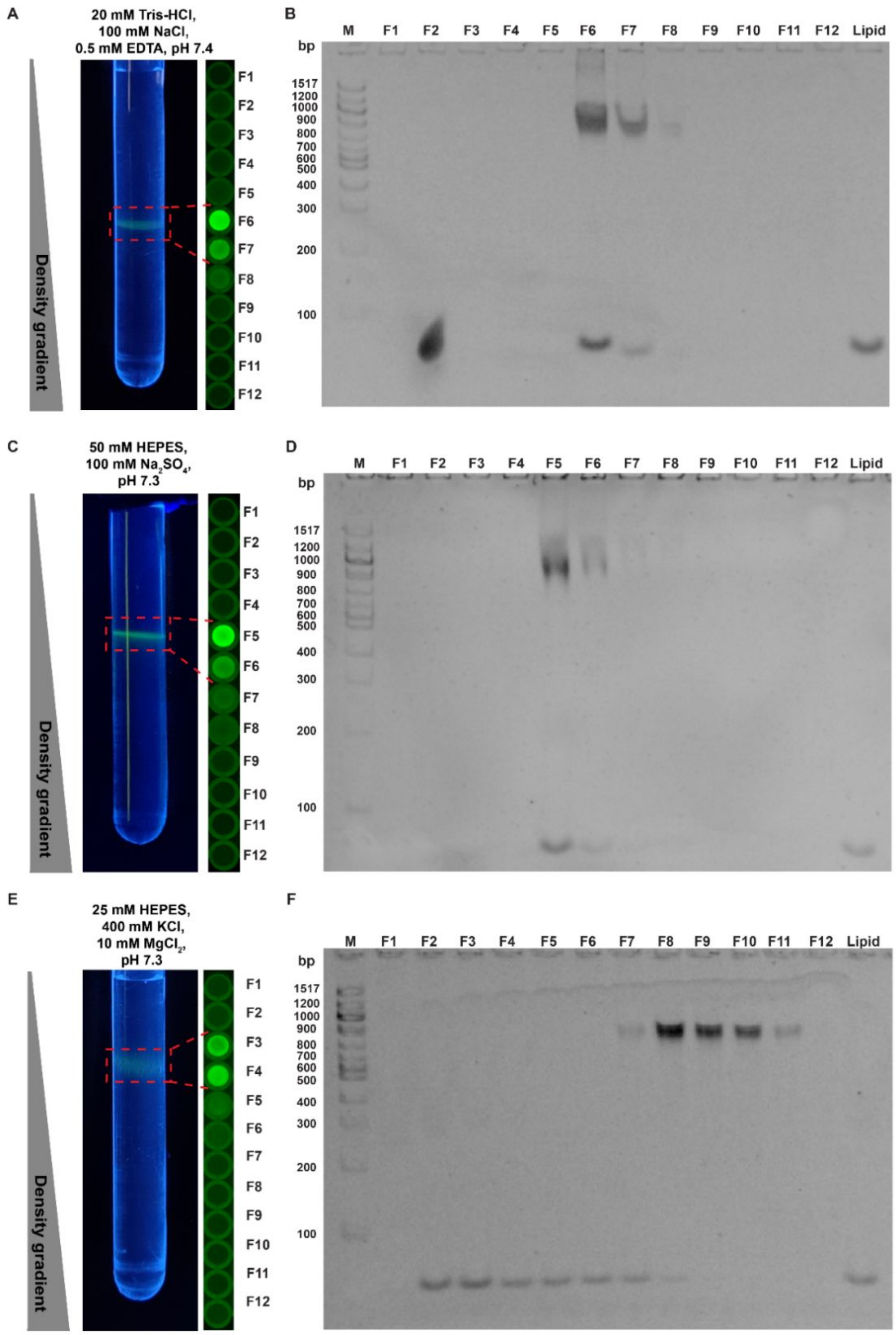


**Figure S14.** Testing buffer conditions. Analysis of DLNs formed with ds-PEG-minicircles (21 x PEG-37) under different buffer conditions. A-D: DLNs form in common buffers containing 100 mM sodium salts as indicated by the colocalization of lipids (bottom band) and DNA (upper band) in fractions 5-7. E-F: Lipids and DNA are not colocalized in the same fractions indicating that DLNs do not form in a high-salt buffers containing 400 mM $K^+$ and 10 mM $Mg^{2+}$ or that they are unstable . See Figure S8 for additional experimental details.

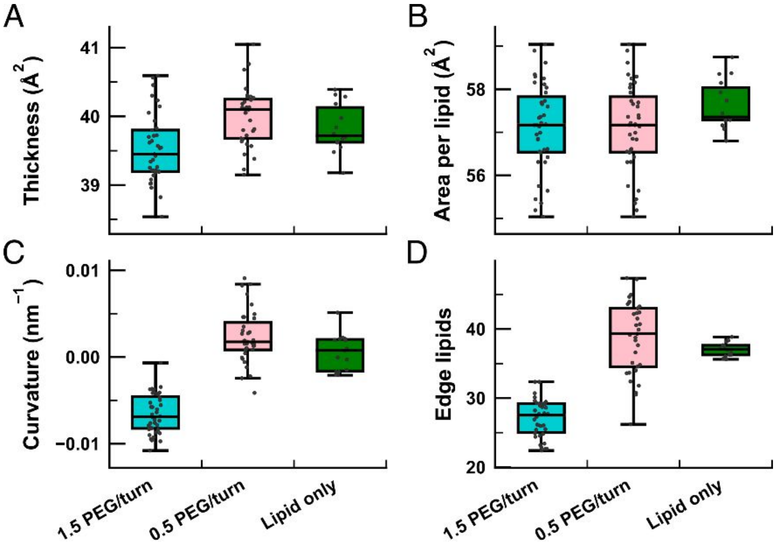


**Figure S15.** Characterization of lipid bilayers in DLNs with 1.5 (cyan) and 0.5 PEG per DNA turn (pink), as well as a lipid-only bilayer (green). Lipid bilayer properties of the central 4-nm radius region of each bilayer, including bilayer thickness (A), area per lipid (B) and curvature (C). (D) Number of lipid headgroups observed along the edges of a DLN and bare bilayer. Here, a lipid is defined to lay along the edge of the bilayer if its C2 atom is located within a 1 nm slab centered on and aligned with the bilayer. Data is shown after the first 250 ns of simulation, including the period of restrained equilibration.

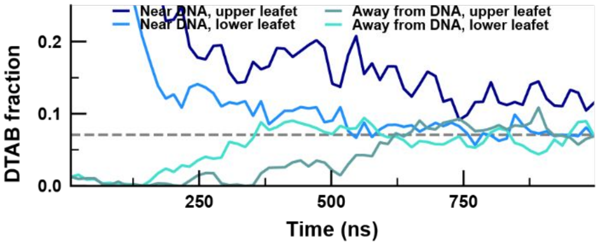


**Figure S16.** DTAB analysis. An analysis of DTAB localization in the lipid bilayer following the same method used for DMTAP (**Error! Reference source not found.**). In the beginning of the simulation, all DTAB molecules were placed at the interface between DNA and lipids. The simulation produces only a modest DTAB enrichment in the upper leaflet near the DNA.

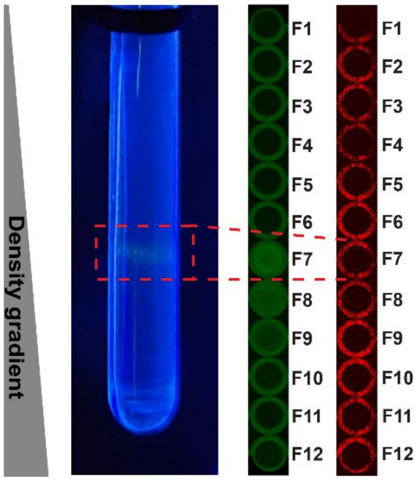


**Figure S17.** Transmembrane peptide reconstitution without PEG. Analysis of a nanodisc reconstitution experiment with lipids, biotinylated TMD peptide and streptavidin-modified quantum dots (QD), but without PEGs on the ds-minicircles. A grainy green fluorescing band in fraction 7 contained lipids in an aggregated state, but no red fluorescing QDs (right wells). In this experiment, QDs were presumably removed along with the peptides in the detergent removal column. This demonstrates that only nanodiscs with a shielding DNA rim can pass the detergent removal column. See Figure S8 for additional experimental details.

# Captions for movies

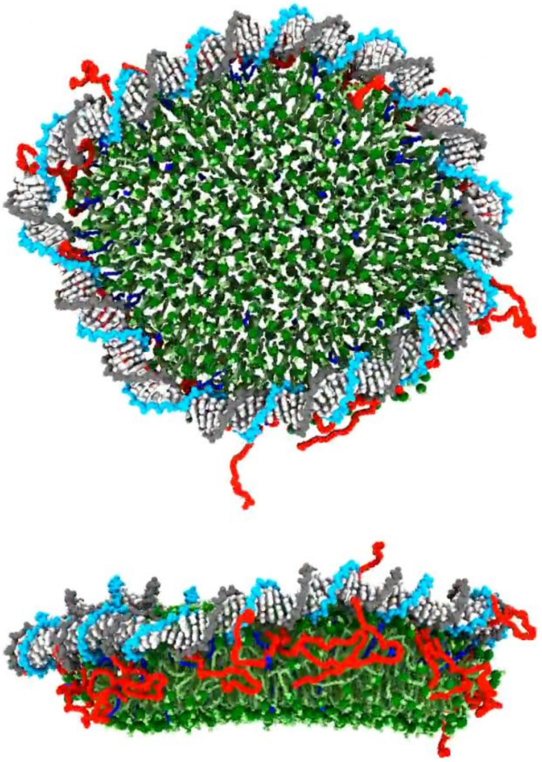

**Movie 1.** All-atom molecular dynamics simulations of PEGylated DLNs. The movie includes the restrained equilibration lasting ~120 ns and subsequent unrestrained simulation lasting nearly 900 ns. A DNA construct (cyan and gray) functionalized with PEG molecules (red) stabilize the lipid bilayer (DMPC, DMTAP and DMPE; green with C2 atom depicted as dark green sphere) doped with DTAP detergent molecules (blue). The top image depicts the DLN from above and the bottom image depicts the same system rotated by 90°.

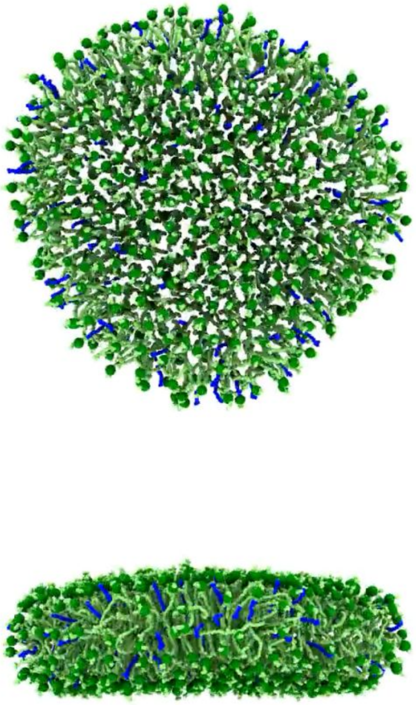

**Movie 2.** All-atom molecular dynamics simulations of a bare lipid bilayer. The movie includes the restrained equilibration lasting ~120 ns and subsequent unrestrained simulation lasting ~400 ns. The system is depicted using the same representations as in **Movie 1**. Note that such a system would be experimentally unstable due to fusion of multiple of these patches into liposomes.

## References


(1) Iric, K.; Subramanian, M.; Oertel, J.; Agarwal, N. P.; Matthies, M.; Periole, X.; Sakmar, T. P.; Huber, T.; Fahmy, K.; Schmidt, T. L. DNA-Encircled Lipid Bilayers. *Nanoscale* **2018**, *10* (39), 18463–18467. https://doi.org/10.1039/C8NR06505E.

(2) Agarwal, N. P.; Matthies, M.; Gür, F. N.; Osada, K.; Schmidt, T. L. Block Copolymer Micellization as a Protection Strategy for DNA Origami. *Angew. Chem. Int. Ed.* **2017**, *56* (20), 5460–5464. https://doi.org/10.1002/anie.201608873.
